# Evaluation of digital binocular therapies for amblyopia after refractive adaptation in treatment-naive children aged 3–13 years: A systematic review and meta-analysis of randomised controlled trials

**DOI:** 10.1371/journal.pone.0361102

**Published:** 2026-10-29

**Authors:** Anju Thadathikunnal Narayanan, David Grant Robinson, J. Margaret Woodhouse

**Affiliations:** 1 Optometry Department, District Hospital Kanhangad, Kanhangad, Kerala, India; 2 School of Optometry and Vision Sciences, Cardiff University, Cardiff, United Kingdom; Aravind Eye Care System, INDIA

## Abstract

**Background:**

Patching has been the mainstay of unilateral amblyopia treatment, but adherence is challenging. Digital binocular therapies have emerged as a novel approach; however, evidence in treatment-naive children remains limited, as existing studies have predominantly included previously treated children.

**Objective:**

To evaluate the effectiveness of digital binocular therapies versus patching for improving visual acuity in treatment-naive children aged 3–13 years with unilateral amblyopia following refractive adaptation.

**Design:**

A systematic review and meta-analysis of randomised controlled trials was conducted in accordance with PRISMA 2020 guidelines and registered in PROSPERO (CRD420261278100). Comprehensive literature searches were conducted from January 2000 to December 2025. Two independent reviewers screened and selected studies, extracted data, and assessed risk of bias using the Cochrane RoB 2 tool. The primary outcome was change in best-corrected visual acuity. Data were pooled using a random-effects model. The GRADE approach was used to rate the certainty of evidence.

**Results:**

Five RCTs (n = 225) were included in the primary analysis. Digital binocular therapies showed no statistically significant difference in visual acuity compared with patching using a random-effects model (MD 0.00 logMAR, 95% CI −0.09 to 0.10; I^2^ = 50.2%). Sensitivity analysis including two additional RCTs judged to be at high risk of bias, one of which compared digital binocular therapy plus spectacles with spectacles alone (7 RCTs; n = 292), yielded similar results (MD 0.04 logMAR, 95% CI −0.03 to 0.10; I^2^ = 75.2%). The certainty of evidence for visual acuity was moderate, downgraded for inconsistency.

**Conclusion:**

Digital binocular therapies may serve as adjunctive treatment options when adherence to patching is challenging. However, current evidence does not demonstrate superiority over patching in treatment-naive children with unilateral amblyopia. The evidence remains limited by the small number of RCTs and modest sample sizes.

**Registration:**

This systematic review and meta-analysis was registered in the International Prospective Register of Systematic Reviews (PROSPERO) under registration number CRD420261278100 on 4 January 2026.

## 1 Introduction

Amblyopia management has evolved significantly over two decades. Landmark studies by the Paediatric Eye Disease Investigator Group (PEDIG) and the MOTAS study established optimal refractive correction as the essential first-line intervention [1–4]. Refractive adaptation alone yields significant visual acuity (VA) gains within 16–18 weeks, with 83% of children stable by 15 weeks [1]. For residual amblyopia, monocular occlusion (patching) or penalisation remains the standard [5,6].

However, patching risks poor compliance, social stigma, and psychological distress [7,8]. As a monocular treatment, it fails to address interocular suppression and binocular function, leading to suboptimal outcomes and potential recurrence [9,10].

Modern interventions target binocular interaction and integration. Early hybrid models combined patching with perceptual learning [11–13], while subsequent treatments like I-BiT and the “Push-Pull” method aimed to reduce interocular suppression via contrast modulation [14,15]. Dichoptic therapy advances this by presenting independent stimuli to each eye to promote balanced input [16,17].

A critical limitation of existing systematic reviews is the inclusion of heterogeneous populations, often children who remained amblyopic after occlusion therapy [18–20]. Such therapeutic history may mask or confound the assessment of the true efficacy of binocular interventions. A review by Asensio-Jurado et al. [21] reported significant within-group improvements in visual acuity and stereoacuity, but no clear superiority over occlusion therapy. Similarly, a 2025 meta‑analysis [22] of 14 RCTs found no significant differences between video‑based dichoptic therapy and patching for BCVA, stereoacuity, or adverse events; subgroup analysis of treatment‑naive children suggested equivalent gains, and a meta‑regression identified adherence >60% as the primary predictor of success. Evidence from four systematic reviews published in 2025 [23–26] reported comparable visual acuity and stereoacuity gains between dichoptic and monocular therapies, though some suggested a marginal advantage for patching [24] and emphasised the influence of amblyopia subtype and treatment duration [23]. Yabanoglu and Taylan Şekeroglu [27] highlighted the potential of binocular approaches to improve binocular function while emphasising the need for larger trials. Digital platforms may improve engagement and reduce treatment burden but have not consistently demonstrated superiority over conventional methods [28]. A recent 2026 systematic review by Martinez-Perez and Oliveira concluded that digital therapies could serve as an adjunctive rather than primary approach in amblyopia management; however, the review included older children and adult populations who were not naive to amblyopia therapy [29].

By strictly restricting this review to treatment‑naive children aged 3–13, immediately following refractive adaptation, we address a significant evidence gap. Isolating children at this early stage allows rigorous assessment of whether digital binocular therapies can serve as a viable first-line alternative to patching. This review evaluates the efficacy of these digital binocular therapies in improving visual outcomes compared with conventional patching, with direct implications for personalised, home‑based clinical strategies.

## 2. Materials and methods

### 2.1 Protocol and registration

The review protocol was prospectively registered with PROSPERO (CRD420261278100) on 4 January 2026. No substantive amendments were made to the review methods or outcomes after PROSPERO registration. The review adhered to the Preferred Reporting Items for Systematic Reviews and Meta-Analyses (PRISMA) 2020 statement [30], and the literature search was conducted and reported using established best-practice methods. Ethical approval was not required because this systematic review used previously published and publicly available data. The Cardiff University Research Ethics Committee confirmed that the study falls under the University’s framework for the ‘Ethical review of research using secondary data and/or publicly available information only’.

### 2.2 Eligibility criteria

The research question was formulated using the Population, Intervention, Comparator, Outcome (PICO) framework recommended in the Cochrane Handbook for Systematic Reviews of Interventions [31].

Population: Children (3–13 years) with unilateral amblyopia following a documented period of refractive adaptation and no other treatment.

Intervention: Digital binocular therapy including dichoptic therapy delivered via gamified platforms such as tablets, virtual reality, or computers, utilising different visual stimuli to each eye to promote binocular interaction and reduce suppression.

Comparator: Refractive adaptation (spectacle correction alone) and/or conventional occlusion therapy (patching).

**Outcomes:** Primary outcome: Change in visual acuity measured using standardised logMAR charts (e.g., ATS HOTV, E-ETDRS).

Secondary outcomes: Stereoacuity, treatment adherence, and adverse events.

#### Inclusion criteria.

Eligible studies were RCTs (2000–2025) involving children aged 3–13, with newly diagnosed amblyopia who completed documented refractive adaptation (≥16–18 weeks or until stable) and received no other amblyopia treatment. Only full-text articles published in English were included, to ensure accurate extraction of complex data, although this may introduce language bias.

Where studies included participants outside the prespecified age range and individual-level data were unavailable to restrict the analysis to the eligible age range, such studies were retained when the majority of participants met the eligibility criteria, in accordance with Cochrane guidance [31].

Of the seven RCTs included in the sensitivity analysis, six compared digital binocular therapy with patching, whereas Iwata et al. [32] compared digital binocular therapy plus spectacles with spectacles alone.

#### Exclusion criteria.

Studies were excluded if they were pilot studies without subsequent follow-up trials, review articles, non-randomised studies, trials without a control group, or interventions involving non-digital therapies (other than spectacles and/or patching). Perceptual learning methods, transcranial magnetic stimulation, acupuncture, purely classical therapies (patching or atropine alone), and combination therapies where crossover prevented estimation of independent treatment effects were also excluded.

One potentially eligible study (Zhu et al. [33]) was excluded because the full text was unobtainable despite contacting the author.

### 2.3 Search strategy

#### Data sources.

A comprehensive literature search was conducted across the following databases: Medline (Ovid), Scopus, Web of Science, Embase, PubMed, Cochrane Library, and Google Scholar. Additional sources included Joanna Briggs Institute, NICE, PROSPERO, SIGN, Trip Database, Centre for Evidence-Based Medicine, and clinical trial registries. Hand-searching and forward/backward reference chaining were performed. Grey literature was explored through open-access university repositories.

A prior search strategy from an earlier version of the review was adapted, refined, and updated to align with the objectives of the current study. The last search was conducted on 20 December 2025. The full search strategy, including keywords, databases searched, search terms, filters, and date ranges, is provided in Supplementary S1 and S2 Tables (S1 File).

#### Study selection and data extraction.

Two reviewers (ATN and J.MW) independently screened titles and abstracts, assessed full-text articles for eligibility, and extracted data using a standardised form. Disagreements at any stage were resolved through discussion or by consultation with a third reviewer (DGR). Statistical analyses and GRADE assessments were performed by DGR. Extracted data are presented in Supplementary S3–S6 Tables (S1 File). The selection process is summarised in the PRISMA 2020 flow diagram of the study selection process (Fig 1) and Supplementary S13 Table (S1 File).

**Fig 1:**
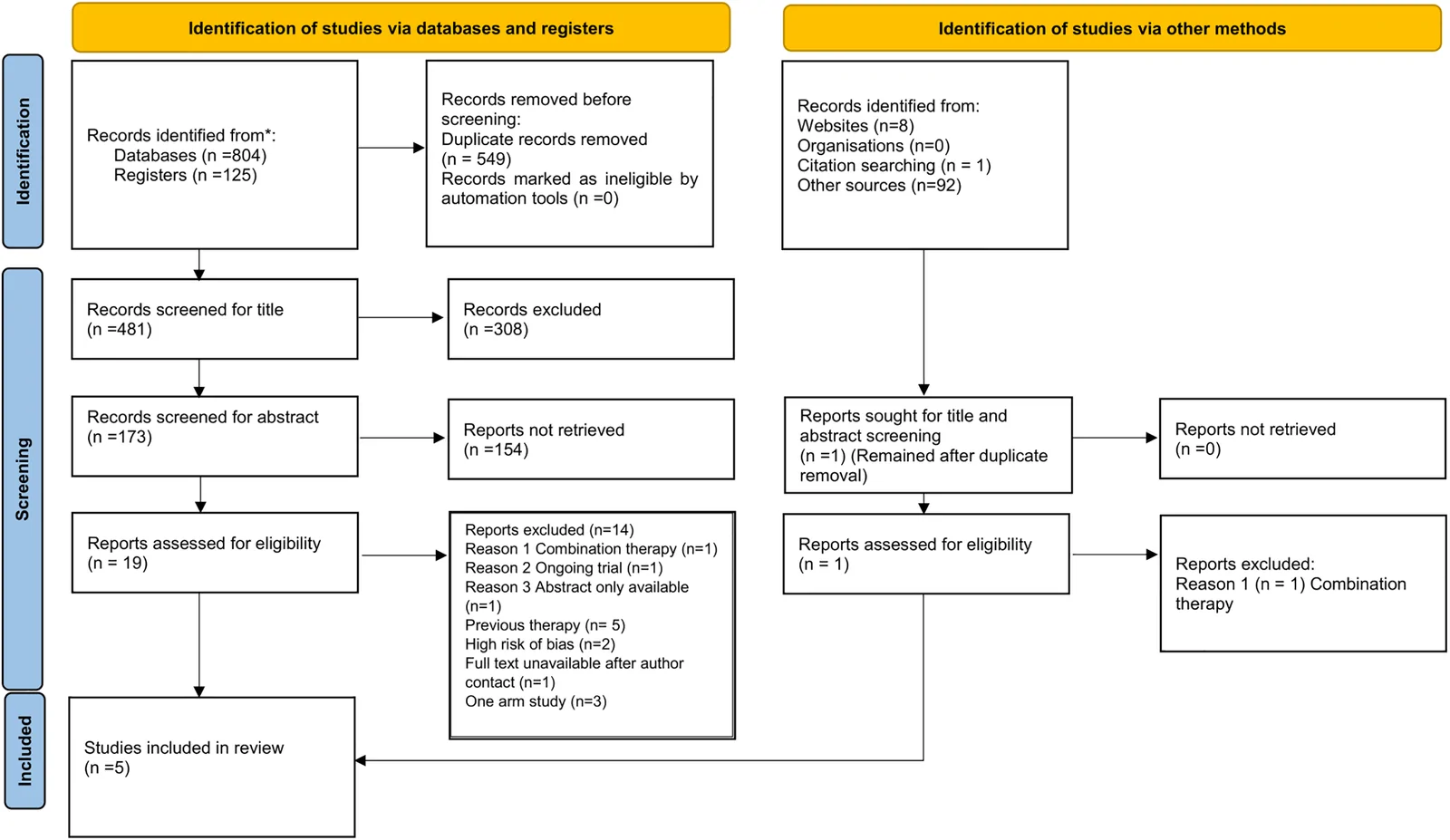
PRISMA 2020 flow diagram showing the study selection process for the systematic review and meta-analysis. Five randomised controlled trials met the eligibility criteria and were included in the primary meta-analysis and GRADE assessment [30].

#### Sample size and data handling.

For each trial, the number of participants randomised at baseline was recorded. For meta-analysis, the effective sample size was defined as the number of participants with available follow-up outcome data. Where intention-to-treat data were unavailable, per-protocol data from participants completing the intervention and outcome assessment were used. Attrition rates and reasons for dropout were documented and considered in the risk of bias assessment. When outcomes were reported as median and interquartile range (IQR) rather than mean and standard deviation (SD), the median was used to approximate the mean, and the SD was estimated as IQR/1.35 assuming a normal distribution [34]. When the SD of change in VA was not reported, the Cochrane‑recommended formula, assuming a correlation coefficient of r = 0.5, was used [31] (Supplementary S5 and S6 Tables (S1 File)).

### 2.4 Risk of bias assessment

Two reviewers independently assessed the risk of bias for each included RCT using the Cochrane Risk of Bias 2 (RoB 2) tool [35]. In accordance with RoB 2 guidance, assessments were conducted at the outcome level for the primary outcome (visual acuity). Each study was evaluated across five domains: bias arising from the randomisation process; deviations from intended interventions; missing outcome data; outcome measurement; and selection of the reported result.

Each domain was judged as ‘low risk’, ‘some concerns’, or ‘high risk’ of bias, leading to an overall risk-of-bias judgement for each outcome. Studies that met the eligibility criteria but were judged to be at high risk of bias were pre-specified for exclusion from the primary meta-analysis and GRADE assessment, but were retained for sensitivity analysis. These assessments informed the certainty of evidence evaluation using the GRADE approach [36].

### 2.5 Certainty of evidence (GRADE)

The certainty of evidence for each outcome was assessed using the GRADE (Grading of Recommendations Assessment, Development and Evaluation) approach [36]. RCTs were initially rated as high-certainty evidence and could be downgraded based on five domains: risk of bias, inconsistency, indirectness, imprecision, and publication bias. Population indirectness was assessed by considering whether differences between the study population and the population of interest were likely to result in a meaningful difference in the relative treatment effect, in accordance with GRADE guidance [37]. The certainty of evidence was categorised as high, moderate, low, or very low. Downgrading by one or two levels was applied depending on the seriousness of concerns identified within each domain.

### 2.6 Data synthesis and statistical analysis

The meta-analysis was performed using the five included studies using R statistical software (R Core Team, 2026) within the RStudio integrated development environment (version 4.6.0) [38,39]. Forest plots were generated using the meta package to visually present the pooled effect estimates and their 95% confidence intervals. Mean differences (MD) in logMAR visual acuity (intervention minus control) were pooled using both fixed-effect and random-effects models. Formal subgroup analyses and meta-regression were not performed because of the small number of included studies. For all studies, pooled estimates were based on change-from-baseline visual acuity values. Heterogeneity was assessed using the I^2^ statistic and the between-study variance (τ^2^). Primary analysis was restricted to studies judged as low risk of bias or with some concerns. Sensitivity analysis included two additional studies at high risk of bias that otherwise met PICO criteria.

Where studies were sufficiently homogeneous in terms of participants, interventions, comparators, outcomes, and methods of measurement, quantitative data were synthesised using meta-analysis. Where substantial clinical or methodological heterogeneity existed, or where outcomes were reported in incompatible formats or with insufficient quantitative data for pooling, a narrative synthesis was undertaken instead. For outcomes unsuitable for meta-analysis, results were summarised in narrative tables following the Synthesis Without Meta-Analysis (SWiM) reporting framework [40].

## 3 Results

### 3.1 Selection of studies

The selection process is detailed in the PRISMA flow diagram (Fig 1). A total of 1,030 records were identified from database searches (n = 804), trial registers (n = 125), and other sources (n = 101). After removing 549 duplicates, 481 records remained for title screening. Title screening excluded 308 records, leaving 173 records for abstract screening. Abstract screening excluded 154 records, resulting in 19 full text reports assessed for eligibility.

Of the 19 full-text reports assessed for eligibility, 14 were excluded. The main reasons were previous amblyopia therapy (n = 5), single-arm design (n = 3), and high risk of bias (n = 2). The remaining exclusions were due to combination therapy (n = 1), an ongoing trial (n = 1), abstract-only publication (n = 1), and unavailability of the full text after contacting the author (n = 1). Two studies (Iwata et al., 2018 and Kadhum et al., 2024 [32,41]) met most of the inclusion criteria but were judged at high risk of bias and were therefore excluded from the primary analysis (they were retained only in the sensitivity analysis). Detailed reasons for exclusion are provided in Supplementary S7 and S8 Tables (S1 File). The remaining five RCTs met all inclusion criteria and were included in the primary meta-analysis and GRADE assessment.

### 3.2 Characteristics of included studies

Five RCTs involving 225 treatment-naive children aged 3–13 years with unilateral amblyopia following refractive adaptation were included [42–46]. For the study by Roy et al. [42], the cohort mean age was 10.5 ± 3.2 years. A subgroup of 24 children older than 12 years was reported separately, with mean ages of 14.0 ± 1.29 years in the video-game group and 13.4 ± 1.12 years in the patching group. Individual-level data were unavailable for children aged ≤13 years and the subgroup represented a small proportion of the total analysed population (24/225; 10.7%). All studies compared digital binocular therapies including dichoptic games, movie-based interventions, or gamified platforms with conventional patching. The primary outcome across studies was change in visual acuity (logMAR) with standard visual acuity tests. Secondary outcomes were stereoacuity, adherence, and adverse events. Additional study details are provided in Table 1.

**Table 1:** Summary of randomised controlled trials comparing digital binocular therapies with their respective control treatments in treatment‑naive children with unilateral amblyopia after refractive adaptation, including two studies at high risk of bias that were excluded from the primary analysis, but included in the sensitivity analysis.*T = intervention group (digital binocular therapy); C = control group (respective comparator). LogMAR = logarithm of the minimum angle of resolution. Log arcsec = logarithm of seconds of arc (stereoacuity), p values for between‑group differences are shown where reported.*

Study and settings	Intervention and control	Age (years) N (T/C) Duration	Mean change¹ in VA logMAR (T/C)	Stereopsis outcome log arcsec (T/C)	Adherence	Adverse events
Holmes et al.² Clinic- based	Falling block games on iPad vs patching	5– < 7 80; 41/39 16 weeks	0.19/0.22	0.22/0³	Not reported	Rare diplopia, 2% skin irritation from patching, and <10% worsening or new-onset heterotropia in both groups
Dahlmann-Noor et al. Home- based	Dichoptic balanced binocular viewing movies on Nintendo 3DS handheld console and patching	3–8 13; 7/6 16 weeks	0.23/0.21	No significant difference reported	BBV Group: Consistently lower adherence mean 56.1%, at 8 weeks; 57.9%, at 16 weeks Occlusion Group: High initial adherence mean 90.1%, at 8 weeks, but dropped substantially by 16 weeks mean 59.2%	Headaches leading to withdrawal in two participants, however this may have been under-reported due to home-based delivery
Chen et al. Home- based	Home-based gamified binocular treatment (GBT, using the Vision Planet Training System) and patching	4–8 37; 16/21 24 weeks	0.19/0.23	0.95/0.82 Mean value reported *p* = 0.71	GBT: Median deviation from target −1.7% (objective device logs; high objective compliance). Patching: Median deviation −13.7% (self-reported logs; significantly worse, p = 0.009).	No adverse events in either arm.
Handa et al. Both clinic and home- based	Occlu-tab, a binocular open-type amblyopia training device and patching	3–12 40; 20/20 8 weeks	0.33/0.16	Not reported	Compliance reported 100% in both groups	No adverse events reported
Roy et al. ⁴ Home- based	Smartphone-based contrast adjusted dichoptic video game	5–15³ 55; 27/28 12 weeks	0.21/0.22	0.10/0.003 Mean ± SD *p* = 0.07	Overall adherence Video game: 55.6% Patching: 78.6% (better) Younger group (5–10 yrs)/Older age group (11–15 yrs) Patching: 94.12%/54.6% Video game: 38.5%/71.5%	Transient adverse events occurred only in the dichoptic group
Iwata et al.^5^ Clinic- based	Occlu-pad with glasses and polarized lenses eye-hand coordination game and spectacles only	3–7 46; 23/23 24 weeks	0.30/0.19	Not reported	Compliance self-reported and non-objective	Not reported
Kadhum et al.⁶ Clinic- based	Supervised dichoptic action video gaming using virtual reality goggles and electronically monitored patching	4–12 21; 7/14 24 weeks	0.30/0.20	1.40/1.40	Intermittent monitoring of compliance reported	Not reported

T– Intervention group.

C– Control group.

^1^Mean change in visual acuity was calculated from baseline and follow-up means where not directly reported in the original publication.

^2^Data for treatment-naive group only.

^3^Median change from baseline to 16 weeks.

^4^Roy et al. enrolled children aged 5–15 years; the analysed cohort had a mean age of 10.5 ± 3.2 years. A separate subgroup of 24 children older than 12 years had mean ages of 14.0 ± 1.29 years in the video-game group and 13.4 ± 1.12 years in the patching group. As individual-level data were unavailable to restrict the analysis to children aged ≤13 years, the trial was retained in the primary analysis, because the subgroup represented a small proportion of the total analysed population (24/225; 10.7%).

^5^High risk of bias; included in sensitivity analysis only (excluded from primary analysis). Iwata et al. compared digital binocular therapy (Occlu-Pad) plus spectacles with spectacles alone rather than patching; the study met the prespecified PICO criteria and was therefore retained for sensitivity analysis.

^6^High risk of bias; included in sensitivity analysis only (excluded from primary analysis).

### 3.3 Individual summaries of included studies

Holmes et al. [43], which included the largest treatment-naive cohort in a clinical setting, found that visual acuity improved in both groups, particularly in younger children, although gains were significantly greater with 2 hours/day of patching. Dahlmann-Noor et al. [44] reported visual acuity outcomes comparable to patching. Chen et al. [45] found small, non-significant differences in visual acuity (*p* = 0.16) and stereoacuity (*p* = 0.71). Although participants in both groups fell short of the prescribed treatment duration, adherence and treatment satisfaction were higher in the binocular group (81.3% vs 60.9%). Handa et al. [46] was the only trial to report a statistically significant between-group advantage favouring digital binocular therapy, whereas the remaining studies reported no significant difference. Roy et al. [42] observed comparable visual acuity improvement at 12 weeks in both groups, although overall compliance was higher with patching; older children, however, showed better adherence to game-based therapy. Additional study details are provided in Table 1, and the strengths and weaknesses of the included studies are summarised in Supplementary S7 and S8 Tables (S1 File).

Table 1: Summary of included trials and studies excluded from the primary analysis because of high risk of bias.

### 3.4 Risk of bias in included studies

Overall, the five included RCTs were judged to have a low risk of bias or some concerns across most domains using the Cochrane RoB 2 tool. Lack of blinding was inherent to the intervention design due to the active nature of digital interventions (domain 2). Additionally, deviations from intended interventions were judged as having some concerns based on potential differences in adherence across groups. However, the risk of bias in other domains remained low, as outcome assessment was generally standardised and evidence of bias in selection of the reported result was minimal. Although masking of participants and personnel was not possible (resulting in ‘some concerns’ in the domain of bias due to deviations from intended interventions), the primary outcome (logMAR visual acuity) was measured objectively by masked assessors using standardised charts; therefore, the risk of bias was not considered serious enough to warrant downgrading the certainty of evidence. Detailed domain-level risk of bias assessments are provided in Supplementary S9 and S10 Tables (S1 File).

### 3.5 Analysis of excluded studies (risk of bias)

Two excluded studies were rated as high risk of bias [32,41]. Iwata et al. [32] was rated to be at high risk of bias due to non-concealed treatment allocation and a lack of assessor masking, introducing significant bias in the randomisation process and measurement of the outcome according to the RoB 2 domains. Methodological reliability was further compromised by the use of Landolt ring charts rather than paediatric-standardised LogMAR protocols (e.g., ATS-HOTV), increasing cognitive demand and measurement inconsistency in younger cohorts. Furthermore, the lack of a matched control for supervised hospital-based training introduced bias due to deviations from intended interventions. The absence of a pre-specified analysis plan and intention-to-treat data suggests a likely overestimation of the reported treatment efficacy.

Kadhum et al. [41] was rated to be at high risk of bias despite a described permuted block randomisation. Significant implementation concerns arose from logistical constraints of clinic-based supervision, leading to a 56% attrition rate in the intervention arm. This created a high risk of bias due to missing outcome data, suggesting that findings likely represent a ‘high-responding’ subset rather than the broader cohort. These concerns were compounded by unmasked assessment and reliance on self-reported compliance.

Summaries of excluded studies are provided in Supplementary S10 Table (S1 File).

### 3.6 Primary outcome

All five included studies reported mean change in best-corrected visual acuity (logMAR) from baseline. Roy et al. [42], Holmes et al. [43], Dahlmann-Noor et al. [44], and Chen et al. [45] demonstrated comparable improvements in visual acuity in the digital binocular therapy and control groups. In contrast, Handa et al. [46] reported a statistically significant greater improvement with digital binocular therapy at 8 weeks.

### 3.7 Primary analysis

Across five trials (n = 225), the pooled analysis showed no clear difference between digital binocular therapies and patching. The random-effect model produced a MD of 0.00 logMAR (95% CI −0.09 to 0.10). The fixed-effect model gave a similar result (MD of −0.01 logMAR (95% CI −0.04 to 0.02). Both models therefore indicate no clear advantage of digital binocular therapy over patching. Moderate heterogeneity was observed (I^2^ = 50.2%; τ^2^ = 0.0023; *p* = 0.09), suggesting moderate variability between studies, although the estimated between-study variance was low. The 95% prediction interval (−0.15 to 0.16 logMAR) crossed zero and extends beyond the clinically meaningful threshold of ±0.1 logMAR [45], indicating that a future study could show a clinically important advantage for either intervention. The results of primary analysis are shown in Fig 2.

**Fig 2:**
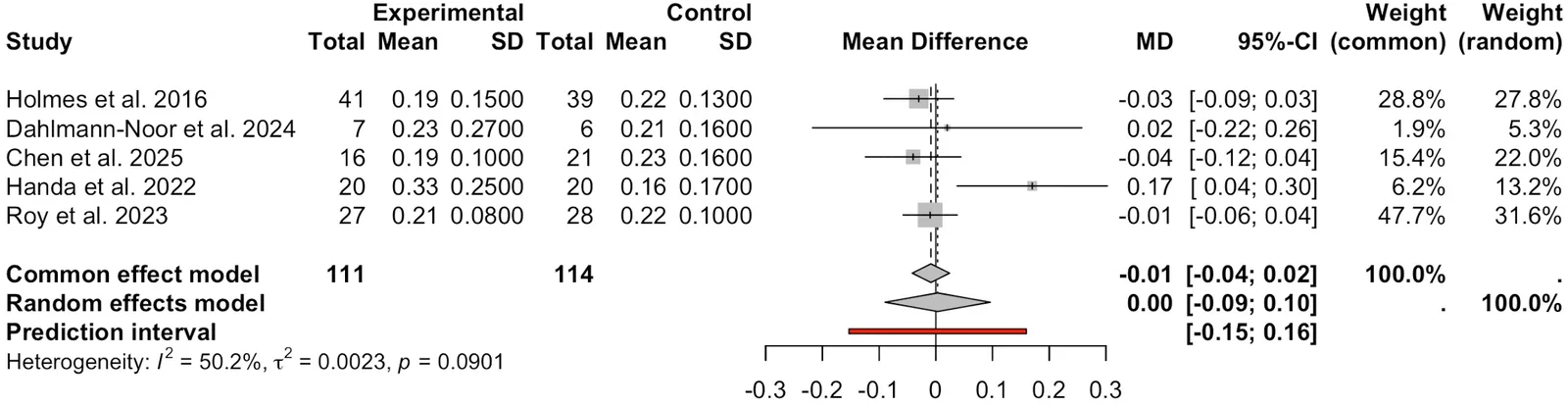
Forest plot of the primary meta-analysis comparing digital binocular therapies with patching for improvement in best-corrected visual acuity (logMAR) in treatment-naïve children with amblyopia after refractive adaptation. The pooled random-effects estimate includes five randomised controlled trials (n = 225).

### 3.8 Sensitivity analysis

When the two high risk of bias studies were added (seven trials, n = 292), the random-effects model showed no statistically significant difference between digital binocular therapies and the respective control treatments in the mean change in best-corrected visual acuity (MD 0.04 logMAR, 95% CI −0.03 to 0.10). The fixed-effect model suggested a small advantage for control treatments (MD 0.03 logMAR, 95% CI 0.00 to 0.06), but this finding was not robust under random-effects modelling and should be interpreted cautiously given the heterogeneity. Heterogeneity increased substantially after inclusion of the high risk-of-bias studies (I^2^ = 75.2%; τ^2^ = 0.0046; *p* = 0.0005), compared with moderate heterogeneity in the primary analysis (I^2^ = 50.2%). Despite this increase, the random-effects pooled estimate remained close to no effect. The 95% prediction interval (−0.15 to 0.22 logMAR) was wide and crossed zero, indicating that the true treatment effect could favour either intervention. Overall, there is no consistent evidence that digital binocular therapies are superior to patching. The results of the sensitivity analysis are shown in Fig 3.

**Fig 3:**
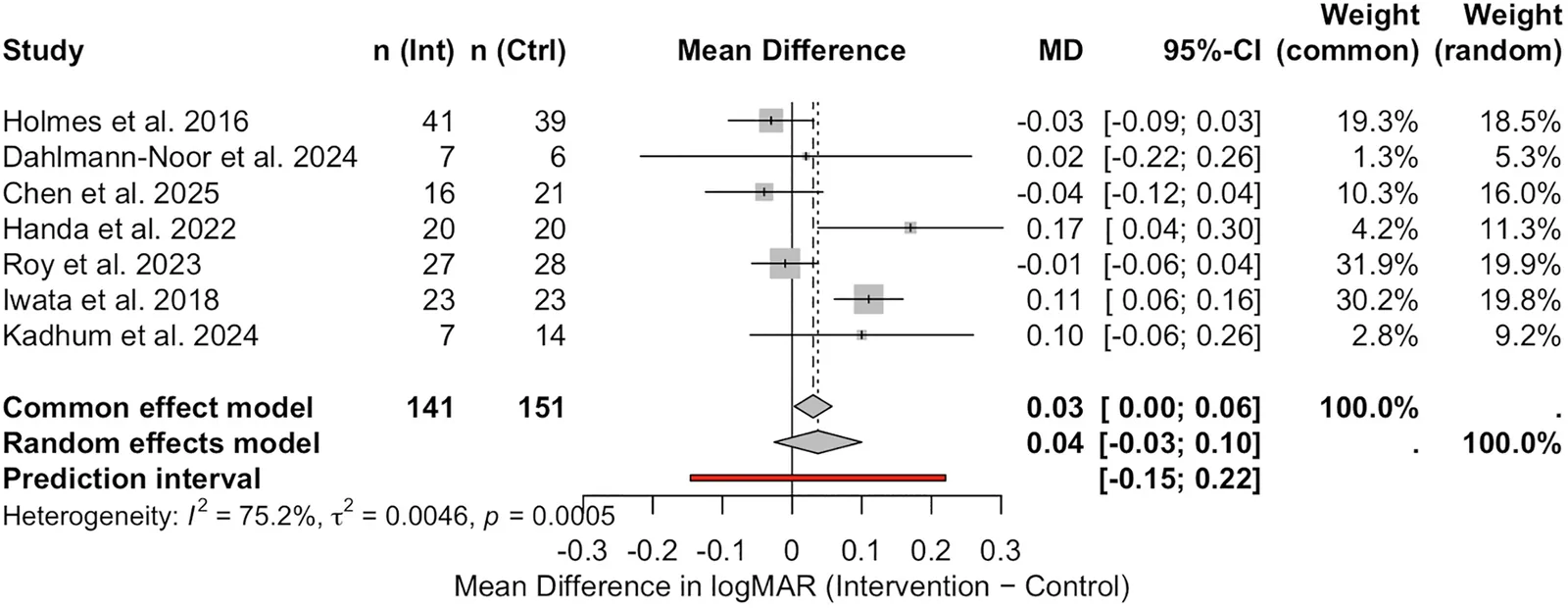
Forest plot of the sensitivity analysis comparing digital binocular therapies with their respective control treatments for improvement in best-corrected visual acuity (logMAR) in treatment-naive children with amblyopia after refractive adaptation. The pooled random-effects estimate includes seven randomised controlled trials (n = 292), including two studies with high risk of bias.

### 3.9 Secondary outcomes

For outcomes unsuitable for meta-analysis due to heterogeneity in measurement methods, reporting formats, and data availability, a structured narrative synthesis was undertaken following the Synthesis Without Meta-analysis (SWiM) reporting guideline, as outlined in the Cochrane Handbook [40]. The SWiM framework provides structured recommendations to improve transparency when statistical pooling was not appropriate. Secondary outcomes were grouped according to outcome domain (stereoacuity, adherence/compliance, and adverse events) and synthesised separately. The direction and consistency of findings were summarised across studies, and the certainty of evidence was assessed using GRADE.

Secondary outcomes (stereoacuity, adherence/compliance, and adverse events) were reported inconsistently across studies, with a mix of median/IQR, mean/SD, qualitative statements, and missing data. Due to the heterogeneous reporting formats, measurement methods, and limited quantitative data, meta-analysis was not feasible. Findings are therefore presented narratively, with GRADE certainty assessments applied to each outcome.

**Stereoacuity:** From the five studies, Holmes et al. [43] reported negligible median changes (binocular 0.22 vs patching 0 log arcsec). Dahlmann-Noor et al. [44] noted no significant change (p value not reported). Chen et al. [45] found no difference (0.95 vs 0.82 log arcsec; *p* = 0.71). Handa et al. [46] did not report stereoacuity. Roy et al. [42] reported no group difference (*p* = 0.07). No statistically significant between-group differences were reported. Overall, reporting was heterogeneous (medians, means, or qualitative statements) and limited (Supplementary S11 Table (S1 File)).

**Adherence/compliance:** Adherence data were sparse and measured using varied methods such as objective device logs, self-reported or not reported. Holmes et al. [43] did not report adherence for the relevant cohort. Dahlmann-Noor et al. [44] reported moderate binocular adherence (mean 56–58%). Chen et al. [45] reported better adherence with binocular therapy (median deviation −1.7% for binocular therapy vs −13.7% for patching), but adherence was measured objectively only in the binocular arm; patching adherence was self‑reported, potentially overestimating compliance. Handa et al. [46] reported both groups as fully compliant, and Roy et al. [42] reported better adherence for the patching group. The findings were heterogeneous in direction and magnitude.

**Adverse events:** Adverse events were rare and mild across the studies. Holmes et al. [43] reported rare diplopia and <10% incidence of new-onset or worsening heterotropia in both groups. Dahlmann-Noor et al. [44] reported headaches and withdrawal of two participants. Chen et al. [45] and Handa et al. [46] reported no serious adverse events. Roy et al. [42] reported transient adverse events in the dichoptic group.

### 3.10 GRADE assessment of secondary outcomes

The certainty of evidence across all three secondary outcomes was rated very low (⊕◯◯◯). Each was downgraded one level for inconsistency (due to variability in measurement methods, reporting, and direction of findings) and two levels for very serious imprecision (small sample sizes, sparse data, and lack of confidence intervals). Although some concerns regarding risk of bias were identified in the measurement of adherence and reporting of adverse events, no further downgrading was applied, as the evidence had already reached very low certainty [37].

#### Stereoacuity and binocular function.

A summary of stereoacuity outcomes across the included studies (excluding those assessed as high risk of bias, namely Iwata et al. [32] and Kadhum et al. [41]) indicated a consistent lack of statistically significant improvement in binocular function following digital intervention. Holmes et al. [43] reported negligible median changes in stereoacuity (binocular 0.22 vs patching 0 log arcsec). Dahlmann-Noor et al. [44] also reported no significant difference in stereopsis recovery. Chen et al. [45] and Roy et al. [42] reported *p* values of 0.71 and 0.07, respectively, indicating that digital therapies did not outperform control treatments. Handa et al. [46] did not report stereoacuity outcomes.

Overall, the evidence for secondary outcomes was limited and of very low certainty. While some studies [42–46] suggested potential advantages of digital binocular therapies in terms of adherence and treatment satisfaction, no reliable conclusions could be drawn regarding stereoacuity, adherence, or adverse events compared with patching.

### 3.11 GRADE analysis

Following quantitative and narrative synthesis (where meta-analysis was not feasible), the certainty of evidence for each outcome was assessed using the GRADE framework across the five domains. The overall certainty ratings are presented in the Summary of Findings table (Table 2). For transparency, the full Cochrane-style GRADE assessments, including domain-level judgments and justifications, are presented in Supplementary S12 Table (S1 File).

**Table 2:** Summary of findings. Summary of findings and GRADE certainty of evidence for the primary outcome (visual acuity) and secondary outcomes (stereopsis, adherence, adverse events) comparing digital binocular therapy with conventional patching in treatment‑naive children aged 3–13 years with unilateral amblyopia after refractive adaptation. *MD = mean difference; CI = confidence interval. GRADE ratings:* ⊕⊕⊕◯ *= moderate certainty;* ⊕⊕◯◯ *= low certainty;* ⊕◯◯◯ *= very low certainty. Downgrading reasons are indicated by superscript numbers (¹, ², ³,) and explained in the footnotes below the table.*

Outcome	Number of participants	Effect estimates	Certainty (GRADE)	Reasons for downgrading
Visual acuity (logMAR)	225 (5 RCTs)	MD 0.00 logMAR (95% CI −0.09 to 0.10)	⊕⊕⊕◯ Moderate ¹	Inconsistency
Stereopsis	225 (5 RCTs)	No significant difference (narrative)	⊕◯◯◯ Very low ²,³	Inconsistency; very serious imprecision
Adherence	225 (5 RCTs)	Heterogeneous findings	⊕◯◯◯ Very low ²,³	Inconsistency; very serious imprecision
Adverse events	225 (5 RCTs)	Rare, mild events	⊕◯◯◯ Very low ²,³	Inconsistency; very serious imprecision

No downgrade was applied for indirectness because the included studies directly addressed the review question with respect to the population, intervention, comparator, and outcomes (PICO). Although one included trial (Roy et al. [42]) reported a subgroup of 24 children older than 12 years, representing 10.7% of the total analysed population (24/225), this was considered insufficient to warrant downgrading for population indirectness, as the subgroup represented a small proportion of the overall analysed population and the overall study population remained broadly consistent with the target population [31,37]. Risk of bias was not downgraded for visual acuity as most included studies were judged as low risk or some concerns, without serious limitations affecting the primary outcome. We did not downgrade visual acuity for imprecision because the 95% confidence interval (−0.09 to 0.10 logMAR) remained largely within the prespecified clinically important threshold of ±0.1 logMAR [47], and was judged sufficiently precise for clinical interpretation. Visual acuity was downgraded one level for inconsistency because moderate statistical heterogeneity was observed (I^2^ = 50.2%), with variation in treatment effects across studies. Although most studies reported similar improvements between groups, one study (Handa et al.) demonstrated a statistically significant benefit favouring digital binocular therapy. The wide prediction interval (−0.15 to 0.16 logMAR) also suggested that the true treatment effect may vary across different study settings. For stereopsis, adherence, and adverse events, downgrading was applied for inconsistency and very serious imprecision, resulting in very low certainty. Publication bias could not be formally assessed due to the small number of included studies (<10). Funnel plot asymmetry and statistical tests for small-study effects are unreliable with this number of trials; therefore, publication bias remains unclear.

1 Downgraded for inconsistency due to moderate heterogeneity, variability in treatment effects across studies and wide prediction interval.
2 Downgraded one level for inconsistency because no quantitative synthesis was possible due to heterogeneity in outcome measurement and reporting, and study findings varied in direction and magnitude of effects.
3 Downgraded two levels for very serious imprecision due to small sample sizes, lack of confidence intervals and uncertain effect estimates.

## 4 Discussion

This systematic review compared digital binocular therapies with conventional patching in treatment-naive children aged 3–13 years with unilateral amblyopia who had completed refractive adaptation and received no prior amblyopia-specific treatment (e.g., patching or atropine). By restricting the population in this way, we minimised confounding from previous therapeutic exposure or carry-over effects, thereby strengthening the internal validity of the findings and enabling evaluation of the true treatment effect of digital binocular therapies following refractive adaptation. It also allowed a direct comparison with patching, which has established benefits but also recognised limitations.

The primary analysis found no clinically meaningful difference in visual acuity improvement between the two approaches (MD 0.00 logMAR; 95% CI −0.09 to 0.10). When the two otherwise eligible RCTs judged to be at high risk of bias [32,41] were added, including one study using spectacle correction alone as the comparator [32], heterogeneity increased substantially (I^2^ = 75.2%), but the pooled estimate remained close to zero without changing the overall conclusion (MD 0.04 logMAR; 95% CI −0.03 to 0.10). These findings suggest that in treatment-naive children, digital binocular therapies have not yet demonstrated superiority over standard patching. The small pooled effect sizes and observed heterogeneity may reflect clinical differences between the trials including participant age, amblyopia subtype, treatment dosage, duration and adherence. Certainty of evidence was moderate for visual acuity and very low for secondary outcomes.

### 4.1 Comparison with existing literature

Our findings are broadly consistent with recent meta-analyses that found no clear advantage of digital therapies over patching [22,23,27]. To our knowledge, no previous systematic review has specifically evaluated digital binocular therapies in treatment-naïve children following refractive adaptation; therefore, direct comparison with previous reviews in this population is not possible. Most of the previous reviews included mixed populations of previously treated and treatment-naive children. Consequently, improvements attributed to digital therapies may partly be confounded by refractive adaptation effects rather than the intervention itself, as shown in some trials (e.g., Manny et al. [48]) conducted in non-naive populations. The key distinction of this review is the focus on treatment-naive children. By isolating children immediately following the 16–18-week refractive adaptation period, we aimed to evaluate the true therapeutic effect, thereby reducing the risk that residual effects of earlier treatment confound the results.

While single-arm feasibility studies [49–51] have reported promising gains in visual acuity and contrast sensitivity, these effects are often observed in highly controlled or supervised settings. Bossi et al. [49] reported a mean amblyopic-eye visual acuity gain of 0.27 ± 0.22 logMAR in 22 treatment-naive children using home-based balanced binocular viewing (BBV) dichoptic movies, with high compliance (89.4% ± 24.2% of prescribed dose) and no significant change in suppression. Gambacorta et al. [50] compared dichoptic gameplay with a combination of patching and gameplay and observed greater acuity improvement in children without recent patching (n = 12, 0.12 ± 0.03 logMAR) compared to those recently patched (n = 6, 0.02 ± 0.05 logMAR; *p* = 0.03), suggesting that prior patching therapy may attenuate response. Liu et al. [51] conducted a pilot single-arm pre–post study demonstrating that dichoptic noise-masked Gabor training produced substantial improvements in maximal tolerable noise contrast (six- to eight-fold) and significantly improved amblyopic-eye acuity (0.15 logMAR in non-patching group; 0.06 logMAR in patching group; both p < 0.001), with stereoacuity gains of 41.6% (*p* = 0.002) and 64.2% (*p* < 0.001), respectively. Contrast sensitivity improved only in the non-patching group (*p* = 0.009), and treatment effects were maintained for up to 10–24 months. These findings suggest that dichoptic demasking may represent a promising binocular approach warranting further controlled trials. However, the absence of concurrent control groups limits the strength of the findings.

The included trials of this review [42–46] reported no statistically significant differences in stereoacuity between binocular therapy and control groups. This lack of improvement in stereoacuity is particularly noteworthy given that these therapies are specifically designed to engage binocular neural pathways. The lack of a clear advantage in visual acuity over patching, coupled with the absence of a demonstrated benefit in stereoacuity despite targeting binocular pathways, suggests that digital binocular therapies may not yet provide clinically meaningful improvements in binocular function. However, the very low certainty of evidence for stereoacuity limits confidence in this finding. Furthermore, secondary outcomes such as adherence and adverse events were inconsistently reported, resulting in very low certainty ratings.

### 4.2 Strengths and limitations

The major strength of this review is the strict inclusion of treatment-naive children after refractive adaptation, minimising confounding and carry-over effects of other amblyopia therapies. Use of the Cochrane RoB 2 tool, GRADE approach, and the SWiM framework further strengthened the transparency and methodological rigour of the review.

Roy et al. [42] included children aged 5–15 years, extending beyond the prespecified upper age limit of 13 years. However, only a small proportion of the total analysed population comprised children older than 13 years (24/225, 10.7%) and individual-level data were unavailable. In accordance with the GRADE [37] and Cochrane guidance [31] studies may be included when the population largely conforms to the prespecified eligibility criteria and the proportion outside the criteria is negligible. This small proportion of participants outside the prespecified age range was not therefore considered sufficient to exclude the study from the primary analysis or to warrant downgrading the certainty of evidence for indirectness.

The principal limitation of the review is the small number of eligible RCTs (n = 5) and the modest overall sample size (n = 225). This has a bearing on the power of this study to quantify the true treatment effect of digital binocular therapies. Moderate heterogeneity (I^2^ = 50.2% in the primary analysis), variation in digital platforms, treatment duration, and adherence were additional limitations. Inclusion of the two high risk-of-bias studies in the sensitivity analysis increased heterogeneity substantially (I^2^ = 75.2%), but did not change the overall conclusion. Meta-analysis of secondary outcomes was not possible because of inconsistent measurement and reporting across studies, necessitating narrative synthesis. Restriction to English-language full-text articles may also have introduced language bias.

The relatively small number of available and included high-quality studies highlight lack of published literature and also obvious gap in the existing evidence base. The scarcity of robust clinical trials suggests that further high-quality research is urgently needed before these interventions can be confidently recommended in routine clinical practice.

### 4.3 Clinical implications and future research

Current evidence does not support replacing patching with digital binocular therapies as first-line treatment in treatment-naive children with unilateral amblyopia following refractive adaptation. Nevertheless, these therapies may have a role as adjunctive or alternative options when adherence to patching or treatment engagement is difficult. Future research must move beyond feasibility towards adequately powered, high-quality RCTs that recruit strictly treatment-naive children. Crucially, trials should incorporate objective adherence monitoring (to distinguish true treatment efficacy from compliance effects), standardise outcome measures (including stereoacuity), systematically report adverse events and include longer-term follow-up. Only then can the field determine whether digital binocular therapies confer meaningful, sustained advantages in both visual and binocular outcomes over conventional patching.

## 5 Conclusion

In treatment-naive children with unilateral amblyopia who had received only spectacle correction, digital binocular therapies demonstrated no statistically significant difference in visual acuity compared with patching. Although designed to target binocular mechanisms like interocular suppression, the certainty of evidence was moderate for visual acuity and very low for secondary outcomes due to modest sample sizes, heterogeneous protocols, and inconsistent secondary outcomes reporting. Consequently, current evidence does not demonstrate superiority over patching in treatment-naive children with unilateral amblyopia following refractive adaptation in routine clinical practice. However, they may represent an alternative or adjunctive treatment option when adherence to patching is challenging. Further high-quality RCTs with larger cohorts, standardised outcomes, and longer follow-up are needed to determine whether digital binocular therapies offer clinically meaningful advantages over conventional amblyopia treatments.

## Supporting information

S1 File Supplementary S1–S13 Tables. (DOCX)
