# Supplementary material for: Evaluation of digital binocular therapies for amblyopia after refractive adaptation in treatment-naive children aged 3–13 years: A systematic review and meta-analysis of randomised controlled trials
Source: PLoS One. 2026 Oct 29;21(10):e0361102. doi: 10.1371/journal.pone.0361102 (PMC13718959; doi:10.1371/journal.pone.0361102)
Supplement: Supplementary file 1 [file pone.0361102.s001.docx]

**S1 Table. Search Terms.**

| S1 | Amblyopi* |
| --- | --- |
| S2 | Child* OR pediatric* OR paediatric* |
| S3 | Dichoptic* |
| S4 | “digital* ther*” |
| S5 | Digital NEXT ther* |
| S6 | “comput* gam*” |
| S7 | Comput* gam* |
| S8 | S1 AND S2 AND S3 |
| S9 | S1 AND S2 AND S4 |
| S10 | S1 AND S2 AND S6 |
| S11 | S8 OR S9 OR S10 |
| S12 | Amblyopi* AND video* |
| S13 | Amblyopi* AND IPAD |
| S14 | Amblyopi* AND television OR TV |
| S15 | Amblyopi* AND movi* |
| S16 | Amblyopi* AND occlu-pad or occlu-tab |
| S17 | Amblyopi* AND naïve |
| S18 | S12 OR S13 OR S14 OR S15 OR S16 OR S17 |
| S19 | S11 AND S18 |

**S2 Table. Search results per databased and registry.**

| **Databases** | **Number of search results** |
| --- | --- |
| Medline (Ovid) | 72 |
| Scopus | 78 |
| Web of Science | 89 |
| Embase (Ovid) | 109 |
| PubMed | 29 |
| Cochrane Library | 139 |
| Google Scholar | 174 |
| Joanna Briggs Institute | 0 |
| NICE | 0 |
| SIGN | 0 |
| Centre for Evidence Based Medicine | 0 |
| Trip Pro | 114 |
| Science Citation Index | 0 |
| ClinicalTrials.gov | 23 |
| WHO International Clinical Trials Registry Platform (ICTRP) | 32 |
| Prospero | 65 |
| EU Clinical Trials Register | 2 |
| ISRCTN registry | 3 |

- **Medline (Ovid)**

| Search | Keyword |  |
| --- | --- | --- |
| S1 | amblyopi* | 10539 |
| S2 | Child* OR pediatric* OR paediatric* | 2867780 |
| S3 | dichoptic* | 1130 |
| S4 | “Digital* ther*” | 19570 |
| S5 | “comput* game*” | 1797 |
| S6 | S1 AND S2 AND S3 | 484 |
| S7 | S1 AND S2 AND S4 | 3510 |
| S8 | S1 AND S2 AND S5 | 770 |
| S9 | S6 OR S7 OR S8 | 892 |
| S10 | S1 AND VIDEO* | 181 |
| S11 | S1 AND IPAD* | 18 |
| S12 | S1 AND Television | 71 |
| S13 | S1 AND movie* | 58 |
| S14 | S1 AND occlu-pad or occlu-tab | 6 |
| S15 | S1 AND treatment-naïve | 7 |
| S16 | S10 OR S11 OR S12 OR S13 OR S14 OR S15 | 223 |
| S17 | S9 AND S16 | 72 |

- **Scopus**

| S1 | Amblyopi* | 10195 |
| --- | --- | --- |
| S2 | Child* OR pediatric* OR paediatric* | 2,506,432 |
| S3 | Dichoptic* | 734 |
| S4 | “digital ther*” | 1782 |
| S5 | “Comput*gam*” | 20456 |
| S6 | S1 AND S2 AND S3 | 89 |
| S7 | S1 AND S2 AND S4 | 38 |
| S8 | S1 AND S2 AND S5 | 29 |
| S9 | S6 or S7 OR S8 | 100 |
| S10 | S1 AND video* | 213 |
| S11 | S1 AND IPAD* | 27 |
| S12 | S1 AND television | 29 |
| S13 | S1 AND movie* | 33 |
| S14 | S1 AND occlu-pad or occlu-tab | 8 |
| S15 | S1 AND treatment-naïve | 7 |
| S16 | S10 OR S11 OR S12 OR S13 OR S14 OR S15 | 285 |
| S17 | S10 AND S16 | 78 |

- **Web of Science**

| S1 | Amblyopi* | 6289 |
| --- | --- | --- |
| S2 | Child* OR pediatric* OR paediatric* | 2,804123 |
| S3 | Dichoptic* | 779 |
| S4 | “digital* ther*” | 1488 |
| S5 | “Comput*gam*” | 9996 |
| S6 | S1 AND S2 AND S3 | 112 |
| S7 | S1 AND S2 AND S4 | 8 |
| S8 | S1 AND S2 AND S5 | 21 |
| S9 | S6 or S7 OR S8 | 118 |
| S10 | S1 AND video* | 195 |
| S11 | S1 AND IPAD* | 128 |
| S12 | S1 AND TELEVISION | 15 |
| S13 | S1 AND movie* | 37 |
| S14 | S1 AND occlu-pad OR occlu-tab | 1 |
| S15 | S1 AND treatment-naïve | 7 |
| S16 | S10 OR S11 0R S12 OR S13 OR S14 OR S15 | 298 |
| S17 | S9 AND S16 | 89 |

- **Embase (Ovid)**

| S1 | Amblyopi* | 16282 |
| --- | --- | --- |
| S2 | Child* OR pediatric* OR paediatric* | 3807823 |
| S3 | Dichoptic* | 1324 |
| S4 | “digital* ther*” | 4119 |
| S5 | “Comput*gam*” | 2438 |
| S6 | S1 AND S2 AND S3 | 155 |
| S7 | S1 AND S2 AND S4 | 13 |
| S8 | S1 AND S2 AND S5 | 22 |
| S9 | S6 or S7 OR S8 | 169 |
| S10 | S1 AND video* | 278 |
| S11 | S1 AND IPAD* | 66 |
| S12 | S1 AND TELEVISION | 56 |
| S13 | S1 AND movie* | 67 |
| S14 | S1 AND occlu-tab or occlu-pad | 7 |
| S15 | S1 AND treatment-naïve | 6 |
| S16 | S10 OR S11 OR S12 OR S13 OR S14 OR S15 | 389 |
| S17 | S9 AND S15 | 109 |

- **PubMed Trials**

| S1 | Amblyopi* | 839 |
| --- | --- | --- |
| S2 | Child* OR pediatric* OR paediatric* | 378556 |
| S3 | Dichoptic* | 67 |
| S4 | “digital* ther*” | 227 |
| S5 | “comput* gam*” | 196 |
| S6 | S1 AND S2 AND S3 | 78 |
| S7 | S1 AND S2 AND S4 | 18 |
| S8 | S1 AND S2 AND S5 | 30 |
| S9 | S6 OR S7 OR S8 | 49 |
| S10 | S1 AND video* | 54 |
| S11 | S1 AND IPAD* | 11 |
| S12 | S1 AND television | 6 |
| S13 | S1 AND movie* | 5 |
| S14 | S1 AND occlu-tab OR occlu-pad | 0 |
| S15 | S1 AND treatment-naïve | 0 |
| S16 | S10 OR S11 OR S12 OR S13 OR S14 OR S15 | 59 |
| S17 | S9 AND S16 | 29 |

- **Cochrane Library**

| S1 | Amblyopi* | 1003 |
| --- | --- | --- |
| S2 | Child* OR pediatric* OR paediatric* | 270660 |
| S3 | Dichoptic* | 121 |
| S4 | Digital ther* | 17669 |
| S54632 | Comput* gam* | 4635 |
| S6 | S1 AND S2 AND S3 | 437 |
| S7 | S1 AND S2 AND S4 | 304 |
| S8 | S1 AD S2 AND S5 | 627 |
| S9 | S6 OR S7 OR S8 | 626 |
| S10 | SI AND video* | 119 |
| S11 | S1 AND IPAD* | 18 |
| S12 | S1 AND television | 7 |
| S13 | S1 AND movie* | 18 |
| S14 | S1 AND occlu-tab OR occlu-pad | 19 |
| S15 | S1 AND treatment-naïve | 1 |
| S16 | S10 OR S11 OR S12 OR S13 OR S14 OR S15 | 281 |
| S17 | S9 AND S16 | 139 |

- **Other databases searched**

| **Grey literature search** | **Keywords** | **Number of search results** |
| --- | --- | --- |
| Arxiv | amblyopi* AND dichoptic AND binocular OR video* AND child* OR comput* OR ipad* OR movi* | 0 |
| Overton | amblyopi* AND dichoptic AND binocular OR video* AND child* OR comput* OR ipad* OR movi* | 73 |
| NDLTD | amblyopi* AND dichoptic AND binocular OR video* AND child* OR comput* OR ipad* OR movi* | 0 |
| OATD | amblyopi* AND dichoptic AND binocular OR video* AND child* OR comput* OR ipad* OR movi* | 19 |

- **Websites reviewed**

<https://www.bynocs.com/>

<https://nova-sight.com/curesight-amblyopia-treatment/>

<https://lazy-i-bit.co.uk/>

<https://www.metacritic.com/game/dig-rush/>

<https://www.prnewswire.com/news/amblyotech-inc./>

<https://tetris.com/play-tetris>

<https://duovision.com/the-lazy-eye-tetris/>

<https://www.seevividly.com/>

<https://www.nyu.edu/research.html>

<https://uwaterloo.ca/research/>

<https://www.auckland.ac.nz/en/fmhs/research.html>

<https://www.aston.ac.uk/research>

**S3 Table. Data extraction table for all included studies.**

| **Title** | *Effect of a Binocular iPad Game vs Part-time Patching in Children Aged 5 to 12 Years with Amblyopia: A Randomized Clinical Trial* | *Feasibility of a new ‘balanced binocular*  *viewing’ treatment for unilateral*  *amblyopia in children aged 3–8 years*  *(BALANCE): results of a phase 2a*  *randomised controlled feasibility trial* | *Comparative effectiveness of gamified binocular treatment versus conventional patching for amblyopia: a randomized clinical trial* | *Comparison of the Effectiveness of Amblyopia Treatment with Eye-patch and Binocular Occlu-tab for the Same Treatment Duration* | *Comparison of Dichoptic Therapy Versus Occlusion Therapy in Children with Anisometropic Amblyopia: A Prospective Randomized Study* |
| --- | --- | --- | --- | --- | --- |
| **Authors** | J.M. Holmes, V. M. Manh, E. L. Lazar, R. W. Beck, E. E. Birch, R. T. Kraker, E. R. Crouch, S. A. Erzurum, N. Khuddus, A. I. Summers, D. K. Wallace. | Dahlmann-Noor AH, John A Greenwood, Andrew Skilton, Daniel Baker, Mohamed Abbas, Emma Clay,  Payal Khandelwal, Denise Dunham, Siobhan Ludden, Amanda Davis, Hakim-Moulay Dehbi, Steven C Dakin. | Chen, Ying  Chen, Yuzhen  Han, Xiao  Yang, Zijian  Ying Chen and Yuzhen Chen contributed equally | Handa, T.  Thakkar, H.  Ramakrishnan, M.  Shah, K.  Prajapati, V.  Sayed, S.  Joshi, A.  Ishigaki, Y. | Roy, S.  Saxena, R.  Dhiman, R.  Phuljhele, S.  Sharma, P. |
| **Published in Journal/Conference** | JAMA Ophthalmology | BMJ Open | Frontiers in Medicine | Indian Journal of Ophthalmology | Journal of Pediatric Ophthalmology & Strabismus |
| **Year** | 2016 | 2023 | 2025 | 2022 | 2023 |
| **Type of Study /**  **Setting** | Multicentre randomised, non-inferiority clinical trial, community and institutional practices | Prospective RCT and data conducted at three sites in UK, interventions administered at home | Prospective, evaluator-masked, single-centre randomized controlled trial (RCT); conducted at Sunshine Rehabilitation Centre, Shanghai, China | Properly randomized, evaluator-masked RCT comparing Occlu-tab (binocular open-type dichoptic device) + glasses vs. glasses + conventional eye patching, India in hospital and home setting. | Prospective, randomized, assessor masked interventional  comparative study, at home setting |
| **Aim** | Non-inferiority analysis of binocular iPad game compared with conventional patching therapy in which new amblyopes with only refractive adaptation and no other amblyopia treatment is extracted from the study included in this review. | To assess the safety of dichoptic balanced binocular viewing (BBV) for amblyopia  in children, adherence, acceptability, and improvement in visual function. | To compare the effectiveness of home-based gamified binocular treatment (GBT, using the Vision Planet Training System) with conventional patching for improving visual acuity (VA) and stereoacuity in children with unilateral amblyopia | The study sought to compare traditional occlusion therapy (eye patching) with the Occlu-tab, a binocular open-type amblyopia training device, in order to assess and contrast their effectiveness in the management of amblyopia. | Evaluate and compare the effectiveness of a smartphone-based dichoptic video game with conventional occlusion therapy in children with anisometropic amblyopia. |
| **Methods** | Falling block games in iPad using red green filter glasses over spectacles to play for 1 hour a day, 7 days a week for 16 weeks. The session could be made shorter but a minimum of 4 days a week to be played for 16 weeks as intervention. The control group was undergoing 2 hour patching daily, 7 days a week for 16 weeks. Shorter sessions with breaks were allowed. | Age-appropriate movies on a Nintendo 3DS handheld console with autostereoscopic display (glasses-free 3D via parallax barrier) for 60 min/day as one or two sessions and occlusion 2 to 6 hours per day.  . | To compare the effectiveness of home-based gamified binocular treatment (GBT, using the Vision Planet Training System) with conventional patching for improving visual acuity (VA) and stereoacuity in children with unilateral amblyopia | Participants were assigned to receive either Occlu-tab binocular open-type training or conventional eye patching. Both interventions were administered for 1 hour per day, three times per week. Visual acuity outcomes were measured at baseline and after 6, 7, and 8 weeks of treatment to evaluate and compare the effectiveness of the two modalities. | Participants were 55 children aged 5–15 years with anisometropic amblyopia, n =27 assigned to video game group and received dichoptic therapy using a contrast-adjusted smartphone game for 2 hours daily, whereas the patching group n = 28 underwent occlusion therapy of the non-amblyopic eye for 6 hours daily. All participants were assessed for best-corrected visual acuity, near vision, contrast sensitivity, and both near and distance stereoacuity at baseline and at 1, 2, and 3 months of follow-up. |
| **Length of Study** | 16 weeks with follow up at 4, 8, 12, and 16 weeks for assessment. | 16 weeks | 6 months treatment + follow-up assessments | 8 weeks | 12 weeks |
| **Participants at the start of trial** | 45 participants in game group and 40 in patching, age 5 to <7. | 13 participants aged 3-8, 7 to BBV and 6 to patching | 42 children enrolled and randomized (GBT: n=20; patching: n=22) | 40 children enrolled, 20 each in each arm | 55 children 27 in game group and 28 in patching |
| **Participants who completed** | T41, C 39. | T 7, C 6 | T 16 C 21 | T 20 C 20 | T 27, C 28 |
| **Age range in years** | 5 <7  Mean age not reported for this subgroup | 3-8  Mean  T 4.81 (0.83)  C 5.13 (0.96) | 4–8 years | 3- 12 years  Mean 8.2 ± 2.2 years | 5–15 years  Mean age 10.5 ± 3.2 years |
| **Inclusion Criteria** | Anisometropic, strabismic, or combined mechanism amblyopic with amblyopic eye visual acuity between 20/40 to 20/200 inclusive, IOD ≥ 3 logMAR lines, fellow eye 2025 or better.  For the subgroup of age 5 to <7 years, no previous amblyopia treatment other than refractive adaptation for at least 16 weeks with stable visual acuity level on two consecutive tests 4 weeks apart.  Eye deviation ≤ 10 prism dioptre, hypermetropia of 3.00D or more, myopia of amblyopic eye of 0.50D or more, astigmatism of 1.50D or more, anisometropia of more than 0.50D spherical equivalent. | Age between 3 and 8 years (inclusive) • Unilateral anisometropic, strabismic or combined mechanism amblyopia (see definitions above) • Best-corrected visual acuity (BCVA) in the amblyopic eye worse than 0.20 logMAR, with a difference between the eyes of 0.20 logMAR or more • Completion of optical treatment prior to inclusion in the trial, if applicable • No prior treatment (including occlusion or atropine), other than optical treatment. | Age 4–8 years  Unilateral amblyopia (strabismus, anisometropia, or both)  ≥2-line interocular VA difference  Amblyopic eye BCVA 20/32 to 20/200  Fellow eye BCVA ≥20/40 (ages 4–5) or ≥20/32 (≥5 years)  Stable VA after ≥8 weeks refractive adaptation | Participants were children aged 3–12 years who presented for the first time and were newly diagnosed with anisometropic amblyopia, defined as an interocular refractive difference ≥ 2.00 D and best-corrected visual acuity (BCVA) of the amblyopic eye ≤ 0.1 logMAR, measured using a Snellen chart by an ophthalmologist or optometrist. Eligible participants were required to attend clinic visits three times per week for 8 weeks following initiation of amblyopia treatment. | Participants were 5 to 15 years of age  Diagnosed with anisometropic amblyopia  Had best corrected visual acuity (BCVA) of 0.2 to 1.0 logMAR in the amblyopic eye  Completed optical adaptation (6 weeks if new glasses were prescribed) |
| **Exclusion Criteria** | Myopia >-6.0 D, severe developmental delay and ocular co-morbidity and systemic anomaly, deviation >10 PD for near, allergic to bandage adhesives, any other amblyopia treatment or prisms in the previous two weeks before enrolling. | Ocular cause for reduced visual acuity other than amblyopia.  Inability/unwillingness to cooperate with the tests.  Developmental disorders that impact adherence. Photosensitive epilepsy, prior ocular surgery, myopia with spherical equivalent greater than -6.0, manifest strabismus greater than 10 prism dioptres with distance or near fixation. | Organic ocular abnormalities  History of ocular surgery or conditions affecting BCVA  Cognitive/communication impairments preventing cooperation | Children were excluded if they had a prior history of amblyopia treatment, were diagnosed with strabismus, were unable to complete visual acuity or refraction assessments, or were already undergoing occlusion therapy with an eye patch. | Previous history of amblyopia treatment  Manifest strabismus  Nystagmus  Corneal opacities Unavailability of an Android smartphone at home |
| **Randomisation** | Via PEDIG website with equal probability to receive either game group or spectacle group, by permuted block design stratified by age group and site, allocation hidden, investigator and participants if masked unclear, outcome assessors are masked. | Parallel-group, phase 2a feasibility randomised controlled trial at two sites, with 1:1 allocation ratio between the two interventions. Allocation hidden, observer masked, but the participants and parents unmasked. | Computer-generated sequence (R software v4.2.0), 1:1 allocation | Randomization based on a permuted block method, performed using random numbers generated in Microsoft Excel, with a 2‑block size. | Participants were randomly assigned into two equal groups using computer-generated random numbers. |
| **Comparison /**  **Control group** | Group 1: Binocular iPad falling block game with red green filters over spectacles  Group 2: Used eye patch with spectacles. | Group 1: Played age-appropriate movies on a Nintendo 3DS handheld console with autostereoscopic display (glasses-free 3D via parallax barrier).  Group 2: Used eye patch | Group 1: Home-based gamified binocular treatment (GBT, using the Vision Planet. Group 2: Conventional patching (90 min daily occlusion of fellow eye, 7 days/week; could split into ≥30 min sessions) | Group 1: Occlu-tab group, children received binocular open-type amblyopia training using the Occlu-tab device for 1 hour per day, three times per week.  Group2: Conventional Eye Patch Group – Children underwent standard occlusion therapy (eye patching) for 1 hour per day, three times per week. | Group 1: Dichoptic Video Game (n = 27)  Played a contrast-adjusted dichoptic Tetris game  2 hours per day, 7 days/week for 3 months  Using red/green anaglyph glasses over spectacles  Group 2: Patching Group (n = 28)  Received occlusion therapy of the non-amblyopic eye  6 hours per day, 7 days/week for 3 months |
| **Follow- Up** | Very minimal loss to follow up and low risk bias (96% of total participants in both groups completed the trial)  From the data extracted for 5 to < 7 age group, it is not reported. | Detailed description of those recruited for the study and those who completed and those who dropped out. High risk drop out affecting conclusiveness of secondary outcomes. | Assessments at 1, 3, and 6 months (±1 week) post-treatment start | Assessments at , 7 and 8 weeks. | Assessed at 1, 2, and 3 months |
| **Adverse Events** | Diplopia very rare, skin irritation 2% in patching group and less than 10% with worsening of existing deviation or onset of new tropia in both groups.  No separate data reported for 5 to < 7 age group. | Headaches leading to withdrawal in two participants, may have been under-reported due to home-based delivery | None reported in either group (no eye strain, headaches, diplopia, worsening VA, or heterotropia increase ≥10 PD). 2 GBT dropouts related to follow-up reluctance (not adverse events) | None reported in both groups | Dichoptic group: Transient side effects – headache, watering, blurred vision, eye pain (46.1% younger, 21.4% older) Relieved by splitting sessions  Patching group: No adverse events reported |
| **Success Criteria** | Participation details were not separately available for the age group selected. For the whole study group, only 22% completed >75% of game in binocular group. It suggests low adherence to game as such. | Best-corrected visual acuity (BCVA): Improved in both arms (standard treatment: 0.47 to 0.26 logMAR; BBV: 0.55 to 0.32 logMAR). No significant between-group difference at 16 weeks.  Interocular balance/suppression- Between-group comparison at 16 weeks (adjusted for baseline): No significant difference (−0.02, 95% CI −0.28 to 0.23, p=0.87). | Not explicitly predefined (efficacy trial); primary: change in amblyopic eye BCVA (logMAR); secondary: stereoacuity change (log arcsec). Equivalence/non-inferiority implied by similar 6-month improvements. | Significant improvement in amblyopic-eye best-corrected visual acuity (BCVA) from baseline. | Improvement in Best Corrected Visual Acuity, contrast sensitivity and near stereoacuity |
| **Results** | See Table 2, Table S12, Figure 2 | See Table 2, Table S12, Figure 2 | See Table 2, Table S12, Figure 2 | See Table 2, Table S12, Figure 2 | See Table 2, Table S12, Figure 2 |
| **Mean amblyopic eye visual acuity improvement** | 0.19 logMAR lines in the binocular group and 0.22 logMAR lines in the patching group. | Binocular group 0.32 logMAR  Patching group 0.26 logMAR; | GBT: 0.19 ± 0.10 logMAR; patching: 0.23 ± 0.16; *p* = 0.156) at 6months similar VA improvement in both groups | Occlu‑tab group (0.33 ± 0.25) significantly greater than that of the eye patch group (0.16 ± 0.17) after 8 weeks of treatment (*P* = 0.02). | Game group BCVA increased to 0.21 ± 0.08 and patching group increased to 0.22 ± 0.10 |
| **Adherence T/C** | Not reported | Occlusion Group: High initial adherence (mean 90.1%, SD 19.7 at 8 weeks), but dropped substantially by 16 weeks (mean 59.2%, SD 24.8).  BBV Group: Consistently lower adherence (mean 56.1%, SD 36 at 8 weeks; 57.9%, SD 30.2 at 16 weeks), with data available for fewer participants (11 at 8 weeks, 8 at 16 weeks). | GBT Median deviation from target -1.7% (objective device logs; high objective compliance).  Patching: Median deviation -13.7% (self-reported logs; significantly worse, p=0.0096).  Higher adherence in GBT linked to faster early gains. | Compliance reported 100% in both groups | Overall compliance:  Video game: 55.6%  Patching: 78.6% (better)  Younger group (5–10 yrs):  Patching: 94.12%  Video game: 38.5%  Older group (11–15 yrs):  Video game: 71.5%  Patching: 54.6% (not significant)  Patching showed better compliance, especially in younger children. |
| **Statistical Analysis** | Mean ± standard deviation, power calculation and sample size estimation, non-inferiority limit and attrition limit set, modified intent-to-treat analysis, analysis of covariance for upper limit of confidence interval, post-hoc analysis. | Mean ±SD (or median ±IQR in case of strong departure from normality) for continuous variables, and count +frequencies  for categorical variables. The endpoints that were continuous in nature, was analysed using analysis of covariance (ANCOVA) and the estimate from ANCOVA (referred to as the ‘treatment effect’) is the additional difference in the BBV intervention  arm compared with the delta (change from baseline) in the occlusion/control arm. Examinations of the within-arm effectiveness of treatments were examined using paired- sample t-tests. A p<0.05 was considered statistically  significant. | Mean ± standard deviation  Mixed-model ANOVA for BCVA changes (time, treatment, interaction; participants as random effect).  Student's t-test for pairwise comparisons.  Wilcoxon signed-rank/non-parametric for stereoacuity.  Bonferroni correction for multiple testing.  Analyses on available data (no imputation specified). | Mean ± standard deviation, power calculation and sample size estimation  One-way Repeated-Measures ANOVA (RM ANOVA) – to compare visual acuity before and after treatment within each group.  Tukey’s post-hoc test – for multiple comparisons following ANOVA.  Independent t-test – to compare the magnitude of visual acuity improvement between the eye-patch and Occlu-tab groups.  Significance level: P < 0.05.  Software used: SigmaPlot (version 12.5, Systat Software, Inc.). | Statistical analysis Repeated measures ANOVA → Within-group comparison (BCVA, near vision, contrast sensitivity)  Two-sample t test → Intergroup comparisons & improvement analysis  Mann–Whitney test → Near & distance stereoacuity (non-parametric data)  Chi-square test → Compliance data  Spearman’s correlation → Correlation analysis  P < 0.05 → Statistically significant |

**S4 Table. Population/Demographics details of the study sample.**

| **Study** | **Age range in years** | **Age group** | **Male/female T/C** | **Sample size**  **T/C** | **Anisometropic/Strabismic/**  **Combined mechanism amblyopia** | **Pre- treatment VA range amblyopic eye/IOD** | **Refractive error range** | **Refractive adaptation** | **Previous amblyopia treatments** |  |
| --- | --- | --- | --- | --- | --- | --- | --- | --- | --- | --- |
| Holmes et al. | 5 - <7  Mean age not reported for this group  7 to 13 | Younger age group  Older age group | Not reported for this subgroup | 80  41/39 | Unavailable for this subgroup | 20/40 to 20/200 inclusive  IOD ≥ 3 logMAR lines | Hypermetropia of 3.00D or more, myopia of amblyopic eye of 0.50D or more, astigmatism of 1.50D or more, anisometropia of more than 0.50D spherical equivalent | 16 weeks and when stable visual acuity at two consecutive checks 4 weeks apart | No previous amblyopia treatment other than spectacles |  |
| Dahlmann-Noor et al. | 3-8 | Younger age group | T 6/10  C 10/6 | 13  7/6 | T 9/4/3  C 8/4/4 | T 0.55 logMAR  C 0.47 logMAR | T 4.93D  C 4.16D | Completion of optical treatment prior to inclusion in the trial | No previous amblyopia treatment other than spectacles |  |
| Chen et al. | 4-8 | Younger age group | Not reported | 37  16/21 | 93%/4.7%/2.3% in both groups | Amblyopic eye VA between 20/32 and 20/200  Age 4-5 BCVA 20/40 or more in dominant eye | No published data available | At least 8 weeks of refractive adaptation with stable visual acuity in the amblyopic eye (changes in VA less than 1 line). | No previous amblyopia treatment other than spectacles |  |
| Handa et al. | 3-12 | Both younger and older age group present | T 17/10  C 18/10 | T 20  C 20 | 100% anisometropic amblyopia | Occlu‑tab groups 20/70 to 20/200 logMAR,  Eye patch 20/80 to 20/125 logMAR | No data available  refraction difference of both eyes ≥ 2 D; | All patients wore complete correction glasses fitted with cycloplegic refraction is the only published data | No previous amblyopia treatment other than spectacles |  |
| Roy et al. | 5-15 | Both younger and older age group present | Not reported | T 27  C 28 | 100% anisometropic amblyopia | Both groups 20/100 range | No data available | 6 weeks of optical adaptation, last refraction should be done within six months | No previous amblyopia treatment other than spectacles |  |
| T-Treatment group  C-Control group  VA-Visual Acuity  IOD-Interocular difference | | | | | | | | | | |

**S5 Table. Data extraction and statistical handling.**

| **Study** | **Change in VA** | **SD of VA change** | **Calculation** |
| --- | --- | --- | --- |
| Holmes et al. | Change in VA available for both groups as logMAR lines in supplementary data. We converted to logMAR | SD of change available from supplementary data |  |
| Dahlmann-Noor et al. | Initial and final VA in both groups given, we find the difference as change | SD of change not available | As the standard deviation (SD) of the change was not reported, it was estimated using the Cochrane-recommended formula for change scores:  $SD_{change}=\sqrt{SD_{baseline}^{2}+SD_{final}^{2}-2r\cdot SD_{baseline}\cdot SD_{final}}$  assuming a correlation coefficient of r = 0.5 between baseline and follow-up measurements. This approach yielded an estimated SD of change of 0.085 logMAR for both treatment groups.[30] |
| Chen et al. | Study gives visual acuity improvement, assumed it is change in VA and used for analysis | SD given |  |
| Handa et al. | Change in VA given | SD given |  |
| Roy et al. | Change in VA given | SD given |  |
| Iwata et al. | Change not given, we calculated it from the final and initial VA | SD not given | As the standard deviation (SD) of the change was not reported, it was estimated using the Cochrane-recommended formula for change scores:  $SD_{change}=\sqrt{SD_{baseline}^{2}+SD_{final}^{2}-2r\cdot SD_{baseline}\cdot SD_{final}}$  assuming a correlation coefficient of r = 0.5 between baseline and follow-up measurements. This approach yielded an estimated SD of change of 0.085 logMAR for both treatment groups.[30] |
| Kadhum et al. | Median VA given, same taken as mean | SD calculated | For Kadhum et al., only median and interquartile range (IQR) were reported. The median was used as an approximation of the mean, and the SD was estimated as IQR / 1.35, assuming a normal distribution [32]. |
| *Abbreviations: VA = visual acuity; SD = standard deviation; IQR = interquartile range. SD imputations were performed as described in the Methods section. All imputed values are clearly flagged in the table.* | | | |

**S6 Table. Final data list.**

| **No** | **Study** | **Duration weeks** | **Ni** | **Mean change in logMAR VA** | **SD** | **NC** | **Mean change in logMAR VA** | **SD** |
| --- | --- | --- | --- | --- | --- | --- | --- | --- |
| 1 | Holmes | 16 | 41 | 0.19 | 0.15 | 39 | 0.22 | 0.13 |
| 2 | Dahlmann | 16 | 7 | 0.23 | 0.27 ᵃ | 6 | 0.21 | 0.16ᵃ |
| 3 | Chen | 24 | 16 | 0.19 | 0.10 | 21 | 0.23 | 0.16 |
| 4 | Handa | 8 | 20 | 0.33 | 0.25 | 20 | 0.16 | 0.17 |
| 5 | Roy | 12 | 27 | 0.21 | 0.08 | 28 | 0.22 | 0.1 |
| 6 | Iwata | 24 | 23 | 0.30 | 0.085 ᵃ | 23 | 0.19 | 0.085 ᵃ |
| 7 | Kadhum | 24 | 7 | 0.30 | 0.15 ᵇ | 14 | 0.20 | 0.22 ᵇ |
| ***ᵃ*** *Standard deviation (SD) of the change was not reported, it was estimated using the Cochrane-recommended formula for change scores:*  $SD_{change}=\sqrt{SD_{baseline}^{2}+SD_{final}^{2}-2r\cdot SD_{baseline}\cdot SD_{final}}$  *Assuming a correlation coefficient of r = 0.5 between baseline and follow-up measurements. This approach yielded an estimated SD of change of 0.085 logMAR for both treatment groups (Cochrane Handbook [30]).*  ***ᵇ*** *Median and interquartile range VA (IQR) were reported. The median was used as an approximation of the mean, and the SD was estimated as IQR / 1.35, assuming a normal distribution [32].*  *VA Visual acuity*  *SD Standard Deviation*  *IQR Interquartile range* | | | | | | | | |

**S7 Table. Strengths and weaknesses of included studies.**

| **Study** | **Intervention T/C, age, duration of trial** | **Strength** | **Weakness** |
| --- | --- | --- | --- |
| **Holmes et al.** | Falling block iPad game/patching  Duration: 16 weeks  Intent-to-treat analysis | This was a multicentre RCT conducted in both institutional and community settings. Allocation was concealed and outcome assessors were masked, indicating low risk of selection and detection bias. Minimal attrition and automated monitoring of play duration and inactivity contributed to low risk of attrition bias. The iPad-based intervention objectively tracked gameplay, with contrast enhancement occurring only when games were played correctly, and session breaks were allowed to accommodate children’s attention spans. Participants could adjust difficulty, improving compliance. The study evaluated binocular therapy as a first-line treatment, including a non-inferiority comparison with conventional patching. | Separate outcome data for children aged 5 to <7 years in our inclusion were not reported and the following are for the whole study. Participants and some investigators were aware of allocation, introducing potential performance and detection bias. Adverse events were limited, with rare diplopia, 2% skin irritation from patching, and <10% worsening or new-onset heterotropia in both groups. No cost analysis was performed, and adherence to red–green goggle use was not objectively monitored. |
| **Dahlmann-Noor et al.** | Free Balance Binocular Viewing using Nintendo 3DS/patching Duration: 16 weeks | This was a Phase 2a feasibility RCT with a clear protocol, 1:1 allocation, and observer-masked outcomes. The innovative glasses-free binocular vision intervention using Nintendo 3DS was well described and customizable. The study captured detailed recruitment (22%), retention (63%), and adherence data, along with rich qualitative feedback. No serious adverse events occurred, and consent/assent procedures were clear. The comparator reflected real-world care, providing valuable pilot data for future Phase 3 trials and highlighting practical barriers such as adherence and integration into daily routines. | The study had a small sample size for completed participants and was primarily a feasibility trial, limiting power for efficacy conclusions. Adherence in the binocular vision group was low (56–58%), and the intervention relied on outdated Nintendo 3DS hardware, discontinued by 2020. The primary outcome (suppression) showed no significant between-group difference and exhibited high variability. COVID-19 disrupted recruitment and study flow, and no patient advisory or public involvement group was formed. Adverse events, including headaches leading to withdrawal in two participants, may have been under-reported due to home-based delivery. Limited demographic diversity was reported, and the study was not powered to detect efficacy. |
| **Chen et al.** | Gamified binocular therapy (Vision Planet) vs. patching  Duration: 24 weeks | This was a properly randomized, evaluator-masked RCT comparing gamified binocular therapy (Vision Planet) with patching. The trial demonstrated faster visual acuity improvement at 1 month (p = 0.0029), with similar visual acuity and stereoacuity gains by 6 months, indicating non-inferiority to patching. Adherence was higher and participant satisfaction greater with the game-based treatment, supported by objective logging. No serious adverse events occurred. The intervention was home-based, engaging, and certified as a medical device in China. The trial was prospectively registered, ensuring transparency and methodological rigor. | The study had a small sample size limiting power and generalizability. Follow-up was short (6 months), so long-term outcomes are unknown. Participants were mostly 4–8-year-olds with mild-to-moderate anisometropic amblyopia, limiting applicability to severe cases, strabismus, older children, or other populations. Treatment dose was unequal (binocular ~180 h vs. patching ~270 h), and patching adherence relied on parent self-report, while binocular adherence was automatically tracked. The single-centre design in China and use of a device developed by the research team may introduce potential bias. |
| **Handa et al.** | Occlu‑tab with conventional eye patching  Duration 8 weeks | This was a properly randomized, evaluator-masked RCT comparing Occlu-tab (a binocular open-type dichoptic device) plus glasses versus glasses plus conventional patching in children with anisometropic amblyopia. The study demonstrated significantly greater visual acuity improvement with Occlu-tab at 8 weeks (0.33 ± 0.25 vs. 0.16 ± 0.17 logMAR; p = 0.02). Treatment duration was identical in both arms (1 hour/day, 3 days/week), and compliance was high. No serious adverse events occurred. The device was home-based, simple to use with an iPad and polarizing film, and did not require additional glasses for the dichoptic effect. | The study had a small multicentre sample and a short follow-up (8 weeks), limiting assessment of long-term visual acuity stability or risk of regression. Stereoacuity outcomes were not reported. Only children with anisometropic amblyopia were included, so effects on strabismic or combined types are unknown. The Occlu-tab device is novel, not widely available, and may introduce potential bias if developed or promoted by the research team, although no conflicts of interest were declared. |
| **Roy et al.** | Dichoptic video  game with adjusted contrast for 2 hours/day and patching  Duration 12 weeks | This was a well-designed prospective RCT with masked outcome assessors, computer-generated randomization, and clear inclusion/exclusion criteria. Comprehensive outcomes—including distance and near BCVA, contrast sensitivity, and near and distance stereoacuity—were assessed at multiple time points. The dichoptic video game group showed significant improvements in contrast sensitivity and near stereoacuity (p = 0.01 and p = 0.006), while visual acuity gains were similar, supporting non-inferiority. The home-based intervention was realistic, with compliance monitored via calendar logs, and adverse effects were transient. The study had a good sample size (n = 55 analysed) and well-balanced baseline characteristics. | The study had a short follow-up (3 months), providing no data on long-term retention or recurrence. Participants and parents were not blinded, introducing high performance bias. Compliance relied on parent-reported calendar logs, with no objective monitoring, and was lower in the video game group (55.6%) than patching (78.6%), particularly in younger children (38.5% vs. 94.1%). The study was single-centre (India), included only children with anisometropic amblyopia aged 5–15 years, and excluded strabismic or mixed cases, limiting generalizability. Long-term safety and recurrence risk were not assessed. |
| T/C -Treatment group/Control group  RCT-Randomised Controlled Trials  VA-Visual Acuity  CS-Contrast Sensitivity | | | |

**S8 Table. Summary of excluded studies.**

| **Study** | **Intervention T/C** | **Study Population** | **Findings** | **Limitations** |
| --- | --- | --- | --- | --- |
| Kadhum et al.  2024  RCT  Netherlands | Supervised dichoptic action video gaming (1 hour/week, supervised by researcher; using virtual reality goggles or similar dichoptic setup for binocular gameplay).  Electronically monitored occlusion therapy (patching, 2 hours/day) | N=21,  aged 4-12,  Per-protocol analysis N=21 | Dichoptic gaming (1 h/week supervised) improved visual acuity (VA) and stereoacuity in children with amblyopia.  Comparable or potentially similar effectiveness to patching (2 h/day), with gaming showing benefits despite much lower treatment time/dose.  Suggests gaming may be efficient (faster or equivalent gains per hour) and engaging; attention/enjoyment could enhance cortical plasticity.  Included in meta-analyses showing binocular/dichoptic approaches often comparable to patching overall.  Mean change in visual acuity in treatment group was 0.30 logMAR and control group was 0.17 logMAR.  Stereoacuity improved significantly in both groups, *p* < 0.001) with no significant difference between the two groups (*p* = 0.609).  Compliance during monitored weeks was 81% for patching and it was ensured whole hours of treatment for the intervention. | Incomplete blinding of outcome assessors, predicting bias in favour of the intervention game group due to detection or expectation bias.  High imbalanced attrition — 56% in gaming vs 18% in occlusion. Likely differential missingness biasing towards better outcomes in gaming completers.  Attrition was highly imbalanced (56% in the gaming group vs. 18% in the patching group), with most dropouts among younger children, including two with small-angle strabismus, likely biasing results toward better outcomes in completers, appears unsuitable as a standard treatment for young children with amblyopia.  Unmasked outcome assessors with detection bias in favour of the intervention group. Intermittent monitoring of patching compliance may have produced a Hawthorne effect, inflating adherence measurements. |
| Iwata et al.  2022  RCT  Japan | Occlu-pad with glasses using white-screen technology and polarized lenses, allowing binocular viewing, eye-hand coordination game for 30 min twice weekly in-hospital for 24 weeks. Control: glasses only. | N=46, aged 3 to 7 | The Occlu-pad treatment group showed a significant improvement in visual acuity compared to the glasses-only group (−0.05 ± 0.08 vs. 0.05 ± 0.09 logMAR; p < 0.0001). Mean improvement was 0.19 logMAR in the Occlu-pad group and 0.30 logMAR in the control group. Compliance with Occlu-pad training was high at 3 months (88.4 ± 18.7%) but declined by 6 months (69.6 ± 19.5%; p = 0.003). No significant correlation was observed between compliance and visual acuity gains at either time point (p = 0.97 at 3 months; p = 0.55 at 6 months), suggesting consistent treatment effect regardless of adherence level. | The study was non-randomized, with treatment allocation based on guardian choice, introducing selection bias and limiting causal inference. Outcome assessors were unmasked, potentially favouring the dichoptic intervention. Visual acuity was measured using a Landolt ring chart rather than paediatric-standardized charts (HOTV or LEA symbols), which may reduce comparability and less optimal in younger children due to higher cognitive demands. Compliance was self-reported and non-objective. The analytic approach was unclear (intention-to-treat vs. per-protocol), further weakening methodological rigor. Absence of a true control group prevents assessment of incremental benefit over optical correction alone, potentially overestimating treatment efficacy. |

**S9 Table. Cochrane risk of bias tool for RCTs.**

Risk of bias was assessed using version 2 of the Cochrane Risk of Bias tool for randomized trials (RoB 2). The tool evaluates bias across five domains using signalling questions, with overall judgements classified as low risk, some concerns, or high risk of bias.

Responses underlined in green are potential markers for low risk of bias, and responses in red are potential markers for a risk of bias. Where questions relate only to sign posts to other questions, no formatting is used.

**Domain 1: Risk of bias arising from the randomization process**

| **Signalling questions** | **Comments** | **Response options** |
| --- | --- | --- |
| 1.1 Was the allocation sequence random? |  | Y / PY / PN / N / NI |
| 1.2 Was the allocation sequence concealed until participants were enrolled and assigned to interventions? |  | Y / PY / PN / N / NI |
| 1.3 Did baseline differences between intervention groups suggest a problem with the randomization process? |  | Y / PY / PN / N / NI |
| Risk-of-bias judgement |  | Low / High / Some concerns |
| Optional: What is the predicted direction of bias arising from the randomization process? |  | NA / Favours experimental / Favours comparator / Towards null /Away from null / Unpredictable |

**Domain 2a: Risk of bias due to deviations from the intended interventions (*effect of assignment to intervention*)**

| **Signalling questions** | **Comments** | **Response options** |
| --- | --- | --- |
| 2.1. Were participants aware of their assigned intervention during the trial? |  | Y / PY / PN / N / NI |
| 2.2. Were carers and people delivering the interventions aware of participants' assigned intervention during the trial? |  | Y / PY / PN / N / NI |
| 2.3. If Y/PY/NI to 2.1 or 2.2: Were there deviations from the intended intervention that arose because of the trial context? |  | NA / Y / PY / PN / N / NI |
| 2.4 If Y/PY to 2.3: Were these deviations likely to have affected the outcome? |  | NA / Y / PY / PN / N / NI |
| 2.5. If Y/PY/NI to 2.4: Were these deviations from intended intervention balanced between groups? |  | NA / Y / PY / PN / N / NI |
| 2.6 Was an appropriate analysis used to estimate the effect of assignment to intervention? |  | Y / PY / PN / N / NI |
| 2.7 If N/PN/NI to 2.6: Was there potential for a substantial impact (on the result) of the failure to analyse participants in the group to which they were randomized? |  | NA / Y / PY / PN / N / NI |
| Risk-of-bias judgement |  | Low / High / Some concerns |
| Optional: What is the predicted direction of bias due to deviations from intended interventions? |  | NA / Favours experimental / Favours comparator / Towards null /Away from null / Unpredictable |

**Domain 2b: Risk of bias due to deviations from the intended interventions (*effect of adhering to intervention*)**

| **Signalling questions** | **Comments** | **Response options** |
| --- | --- | --- |
| 2.1. Were participants aware of their assigned intervention during the trial? |  | Y / PY / PN / N / NI |
| 2.2. Were carers and people delivering the interventions aware of participants' assigned intervention during the trial? |  | Y / PY / PN / N / NI |
| 2.3. [If applicable:] If Y/PY/NI to 2.1 or 2.2: Were important non-protocol interventions balanced across intervention groups? |  | NA / Y / PY / PN / N / NI |
| 2.4. [If applicable:] Were there failures in implementing the intervention that could have affected the outcome? |  | NA / Y / PY / PN / N / NI |
| 2.5. [If applicable:] Was there non-adherence to the assigned intervention regimen that could have affected participants’ outcomes? |  | NA / Y / PY / PN / N / NI |
| 2.6. If N/PN/NI to 2.3, or Y/PY/NI to 2.4 or 2.5: Was an appropriate analysis used to estimate the effect of adhering to the intervention? |  | NA / Y / PY / PN / N / NI |
| Risk-of-bias judgement |  | Low / High / Some concerns |
| Optional: What is the predicted direction of bias due to deviations from intended interventions? |  | NA / Favours experimental / Favours comparator / Towards null /Away from null / Unpredictable |

**Domain 3: Missing outcome data**

| **Signalling questions** | **Comments** | **Response options** |
| --- | --- | --- |
| 3.1 Were data for this outcome available for all, or nearly all, participants randomized? |  | Y / PY / PN / N / NI |
| 3.2 If N/PN/NI to 3.1: Is there evidence that the result was not biased by missing outcome data? |  | NA / Y / PY / PN / N |
| 3.3 If N/PN to 3.2: Could missingness in the outcome depend on its true value? |  | NA / Y / PY / PN / N / NI |
| 3.4 If Y/PY/NI to 3.3: Is it likely that missingness in the outcome depended on its true value? |  | NA / Y / PY / PN / N / NI |
| Risk-of-bias judgement |  | Low / High / Some concerns |
| Optional: What is the predicted direction of bias due to missing outcome data? |  | NA / Favours experimental / Favours comparator / Towards null /Away from null / Unpredictable |

**Domain 4: Risk of bias in measurement of the outcome**

| **Signalling questions** | **Comments** | **Response options** |
| --- | --- | --- |
| 4.1 Was the method of measuring the outcome inappropriate? |  | Y / PY / PN / N / NI |
| 4.2 Could measurement or ascertainment of the outcome have differed between intervention groups? |  | Y / PY / PN / N / NI |
| 4.3 If N/PN/NI to 4.1 and 4.2: Were outcome assessors aware of the intervention received by study participants? |  | NA / Y / PY / PN / N / NI |
| 4.4 If Y/PY/NI to 4.3: Could assessment of the outcome have been influenced by knowledge of intervention received? |  | NA / Y / PY / PN / N / NI |
| 4.5 If Y/PY/NI to 4.4: Is it likely that assessment of the outcome was influenced by knowledge of intervention received? |  | NA / Y / PY / PN / N / NI |
| Risk-of-bias judgement |  | Low / High / Some concerns |
| Optional: What is the predicted direction of bias in measurement of the outcome? |  | NA / Favours experimental / Favours comparator / Towards null /Away from null / Unpredictable |

**Domain 5: Risk of bias in selection of the reported result**

| **Signalling questions** | **Comments** | **Response options** |
| --- | --- | --- |
| 5.1 Were the data that produced this result analysed in accordance with a pre-specified analysis plan that was finalized before unblinded outcome data were available for analysis? |  | Y / PY / PN / N / NI |
| Is the numerical result being assessed likely to have been selected, on the basis of the results, from... |  |  |
| 5.2. ... multiple eligible outcome measurements (e.g. scales, definitions, time points) within the outcome domain? |  | Y / PY / PN / N / NI |
| 5.3 ... multiple eligible analyses of the data? |  | Y / PY / PN / N / NI |
| Risk-of-bias judgement |  | Low / High / Some concerns |
| Optional: What is the predicted direction of bias due to selection of the reported result? |  | NA / Favours experimental / Favours comparator / Towards null /Away from null / Unpredictable |

**Overall risk of bias**

| Risk-of-bias judgement | Low / High / Some concerns |
| --- | --- |
| Optional: What is the overall predicted direction of bias for this outcome? | NA / Favours experimental / Favours comparator / Towards null /Away from null / Unpredictable |

**S10 Table. Cochrane Risk of bias assessment of all the studies taken for critical appraisal.**

Assessed using the Cochrane Risk of Bias 2 tool across five domains, with overall judgements rated as low risk, some concerns, or high risk of bias.

**Domain 1: Risk of bias arising from the randomization process**

| **Signalling questions** | **Holmes et al.** | **Kadhum et al.** | **Dahlmann-Noor et al.** | **Chen et al.** | **Iwata et al.** | **Handa et al.** | **Roy et al.** |
| --- | --- | --- | --- | --- | --- | --- | --- |
| 1.1 Was the allocation sequence random? | Y | Y | Y | Y | N | Y | Y |
| 1.2 Was the allocation sequence concealed until participants were enrolled and assigned to interventions? | Y | NI | Y | Y | N | NI | NI |
| 1.3 Did baseline differences between intervention groups suggest a problem with the randomization process? | N | N | N | N | N | N | N |
| Risk-of-bias judgement | Low | Some concerns under allocation concealment | Low | Low | High | Some concerns under unclear allocation and no statement on baseline balance | Some concerns under allocation concealment |
| Optional: What is the predicted direction of bias arising from the randomization process? | NA  no bias predicted | Unpredictable no strong baseline imbalance to suggest | NA  no bias predicted | NA-randomization appears reliable/no bias direction predicted | Favours experimental  No true randomization or allocation concealment, reducing internal validity compared to RCTs.  Potential confounding (e.g., more motivated/compliant families choosing dichoptic, inflating its apparent benefit). | Unpredictable  No baseline data that suggest direction | Unpredictable no strong baseline imbalance to suggest |

**Domain 2a: Risk of bias due to deviations from the intended interventions (effect of assignment to intervention)**

| **Signalling questions** | **Holmes et al.** | **Kadhum et al.** | **Dahlmann-Noor et al.** | **Chen et al.** | **Iwata et al.** | **Handa et al.** | **Roy et al.** |
| --- | --- | --- | --- | --- | --- | --- | --- |
| 2.1. Were participants aware of their assigned intervention during the trial? | Y | Y | Y | Y | Y | Y | Y |
| 2.2. Were carers and people delivering the interventions aware of participants' assigned intervention during the trial? | Y | Y | Y | Y | Y | Y | Y |
| 2.3. If Y/PY/NI to 2.1 or 2.2: Were there deviations from the intended intervention that arose because of the trial context? | N/PN | N/PN | N/PN | PN | PN | NI | N/PN |
| 2.4 If Y/PY to 2.3: Were these deviations likely to have affected the outcome? | NA | NA | NA | NA | NA | NA | NA |
| 2.5. If Y/PY/NI to 2.4: Were these deviations from intended intervention balanced between groups? | NA | NA | NA | NA | NA | NA | NA |
| 2.6 Was an appropriate analysis used to estimate the effect of assignment to intervention? | Y | Y | Y | Y | Y | Y | Y |
| 2.7 If N/PN/NI to 2.6: Was there potential for a substantial impact (on the result) of the failure to analyse participants in the group to which they were randomized? | NA | NA | NA | NA | NA | NA | NA |
| Risk-of-bias judgement | Some concerns, blinding, moderate non-adherence but handled | Some concerns about blinding | Some concerns about blinding | Some concerns about blinding | Some concerns about blinding | Some concerns about blinding | Some concerns about blinding |
| Optional: What is the predicted direction of bias due to deviations from intended interventions? | Unpredictable  Non-adherence could bias towards null; expectation for "fun" game could favour binocular — no dominant effect. | Unpredictable (High dropout in gaming may bias towards null; expectation for supervised gaming could favour it — no dominant effect. | Unpredictable  Non-adherence similar in both groups may bias towards null; expectation for novel BBV could favour it — no dominant effect. | Unpredictable  Non-adherence likely towards null; expectation bias possibly favours gaming; no clear dominant effect. | Unpredictable  Non-adherence could bias towards null; expectation for "fun" game could favour binocular — no clear dominance. | Unpredictable Non-adherence could bias towards the null; expectation bias could favour Occlu-tab; no clear dominance due to lack of adherence data. | Unpredictable  Non-adherence likely towards null; expectation bias possibly favours gaming; no clear dominant effect. |

**Domain 2b: Risk of bias due to deviations from the intended interventions (*effect of adhering to intervention*)**

| **Signalling questions** | **Holmes et al.** | **Kadhum et al.** | **Dahlmann-Noor et al.** | **Chen et al.** | **Iwata et al.** | **Handa et al.** | **Roy et al.** |
| --- | --- | --- | --- | --- | --- | --- | --- |
| 2.1. Were participants aware of their assigned intervention during the trial? | Y | Y | Y | Y | Y | Y | Y |
| 2.2. Were carers and people delivering the interventions aware of participants' assigned intervention during the trial? | Y | Y | Y | Y | Y | Y | Y |
| 2.3. [If applicable:] If Y/PY/NI to 2.1 or 2.2: Were important non-protocol interventions balanced across intervention groups? | NI | NI | NI | NI | NI | NI | NI  no mention of additional non-protocol treatments |
| 2.4. [If applicable:] Were there failures in implementing the intervention that could have affected the outcome? | N/PN | N/PN | N/PN | N/PN | PN | N/PN | N/PN |
| 2.5. [If applicable:] Was there non-adherence to the assigned intervention regimen that could have affected participants’ outcomes? | N | **Y** | Y | N | N | N | **Y**  very low compliance in younger age group (38.5%) and subjective nature of compliance tracking |
| 2.6. If N/PN/NI to 2.3, or Y/PY/NI to 2.4 or 2.5: Was an appropriate analysis used to estimate the effect of adhering to the intervention? | Y | N | N | Y | Y | Y | N |
| Risk-of-bias judgement | Some concerns about awareness/non-blinding + differential non-adherence (favouring supervised arm) without adjustment for adherence effects | Some concerns about awareness/non-blinding + differential non-adherence (favouring supervised arm) without adjustment for adherence effects. | Some concerns about awareness/non-blinding + differential non-adherence (favouring supervised arm) without adjustment for adherence effects | Some concerns about blinding | Non-blinding/awareness of interventions + | Some concerns regarding awareness/non-blinding | Some concerns about awareness/non-blinding, subjective compliance tracking and non-adherence particularly in younger age group |
| Optional: What is the predicted direction of bias due to deviations from intended interventions? | Unpredictable  Non-adherence (more in binocular group) may bias towards null; expectation bias may favour binocular; no dominant effect. | Unpredictable  Non-adherence biases towards null; expectation bias favours experimental; no dominant effect. | Unpredictable  Non-adherence may bias towards null; expectation bias may favour BBV; no dominant effect. | Unpredictable  Non-adherence likely biases towards null; expectation bias may favour binocular group; no dominant effect | Unpredictable  Non-adherence may bias towards null; expectation bias may favour Occlu-Pad; no dominant effect. | Unpredictable  expectation bias may favour occlu-tab. | Unpredictable  Lower compliance of experimental group pull the result towards null, expectation bias may favour phone-based video games, adherent children showed better results, proves lack of adherence as main driver of bias |

**Domain 3: Missing outcome data**

| **Signalling questions** | **Holmes et al.** | **Kadhum et al.** | **Dahlmann-Noor et al.** | **Chen et al.** | **Iwata et al.** | **Handa et al.** | **Roy et al.** |
| --- | --- | --- | --- | --- | --- | --- | --- |
| 3.1 Were data for this outcome available for all, or nearly all, participants randomized? | Y | **N** | **N** | **Y** | **Y** | **Y** | **Y**  Data from 55 out of 60 were analysed |
| 3.2 If N/PN/NI to 3.1: Is there evidence that the result was not biased by missing outcome data? | NA | N | N | NA | NA | NA | NA |
| 3.3 If N/PN to 3.2: Could missingness in the outcome depend on its true value? | NA | PY | PY | NA | NA | NA | NA |
| 3.4 If Y/PY/NI to 3.3: Is it likely that missingness in the outcome depended on its true value? | NA | PY | PY | NA | NA | NA | NA |
| Risk-of-bias judgement | Low risk, good retentions, drop out minimal and balanced | High imbalanced attrition — 56% in gaming vs 18% in occlusion. Likely differential missingness biasing towards better outcomes in gaming completers.) | Some concerns  (Moderate-high attrition, some differential missingness, but feasibility trial context and reasons reported.) | Low | Low | Low | Low |
| Optional: What is the predicted direction of bias due to missing outcome data? | NA / Not applicable (low risk of bias in this domain |  | Unpredictable (Missingness may favour completers in both arms — unclear direction.) | NA / Not applicable (low risk of bias in this domain – no meaningful missing data issue to predict direction). | NA / Not applicable (low risk of bias in this domain | NA / Not applicable (low risk of bias in this domain | NA / Not applicable (low risk of bias in this domain |

**Domain 4: Risk of bias in measurement of the outcome**

| **Signalling questions** | **Holmes et al.** | **Kadhum et al.** | **Dahlmann-Noor et al.** | **Chen et al.** | **Iwata et al.** | **Handa et al.** | **Roy et al.** |
| --- | --- | --- | --- | --- | --- | --- | --- |
| 4.1 Was the method of measuring the outcome inappropriate? | **N** | **N** | **N** | **N** | **Y** | **N** | **N** |
| 4.2 Could measurement or ascertainment of the outcome have differed between intervention groups? | **N** | **N** | **N** | **N** | **N** | **N** | **N** |
| 4.3 If N/PN/NI to 4.1 and 4.2: Were outcome assessors aware of the intervention received by study participants? | N | NI | N | N | NI | N | N |
| 4.4 If Y/PY/NI to 4.3: Could assessment of the outcome have been influenced by knowledge of intervention received? | NA | PY | NA | NA | PY | NA | NA |
| 4.5 If Y/PY/NI to 4.4: Is it likely that assessment of the outcome was influenced by knowledge of intervention received? | NA | PY | NA | NA | PY | NA | NA |
| Risk-of-bias judgement | Low | High  No assessor masking | Low | Low | High  No assessor blinding reported | Low | Low |
| Optional: What is the predicted direction of bias in measurement of the outcome? | No bias concerns, no predicted direction/NA | Incomplete blinding of outcome assessors, predicting bias in favour of the supervised dichoptic gaming group (potential overestimation of its VA efficiency and improvement relative to monitored occlusion due to detection/expectation bias. | No bias concerns, no predicted direction/NA | No bias concerns, no predicted direction/NA | Unmasked outcome assessors, predicting bias in favor of the dichoptic polarizing film group, potential overestimation of its VA benefit relative to patching due to detection/expectation bias. | No bias concerns, no predicted direction/NA | No bias concerns, no predicted direction/NA |

**Domain 5: Risk of bias in selection of the reported result**

| **Signalling questions** | **Holmes et al.** | **Kadhum et al.** | **Dahlmann-Noor et al.** | **Chen et al.** | **Iwata et al.** | **Handa et al.** | **Roy et al.** |
| --- | --- | --- | --- | --- | --- | --- | --- |
| 5.1 Were the data that produced this result analyzed in accordance with a pre-specified analysis plan that was finalized before unblinded outcome data were available for analysis? | Y | Y | Y | Y | NI | Y | Y |
| Is the numerical result being assessed likely to have been selected, on the basis of the results, from... |  |  |  |  |  |  |  |
| 5.2. ... multiple eligible outcome measurements (e.g. scales, definitions, time points) within the outcome domain? | N | N | N | N | N | N | N |
| 5.3 ... multiple eligible analyses of the data? | N | N | N | N | N | N | N |
| Risk-of-bias judgement | Low risk | Low risk | Low risk (Prospective registration, pre-specified plan.) Over all some concerns | Low | Low  All planned outcomes reported; no selective reporting evident. | Low risk | Low risk |
| Optional: What is the predicted direction of bias due to selection of the reported result? | NA no bias direction to predict |  |  | NA no bias direction to predict | NA  No evidence of selective outcome or analysis selection based on results. No bias direction to predict | NA no bias direction to predict | NA no bias direction to predict |

**Overall risk of bias**

| **Risk-of-bias judgement** | **Holmes et al.**  **Low** | **Kadhum et al.**  **High** | **Dahlmann-Noor et al.**  **Low** | **Chen et al.**  **Low** | **Iwata et al.**  **High** | **Handa et al.**  **Low** | **Roy et al.**  **Low** |
| --- | --- | --- | --- | --- | --- | --- | --- |
| Optional: What is the overall predicted direction of bias for this outcome? |  | Incomplete blinding of outcome assessors, predicting bias in favour of the intervention game group due to detection or expectation bias |  |  | Improper randomisation favouring experimental group as parents’ choice to select treatment.  Unmasked outcome assessors, predicting bias in favour of the dichoptic polarizing film due to detection or expectation bias |  |  |

**S11 Table. Improvement in stereoacuity.**

| **Study** | **Intervention group** | **Median Stereopsis in logarcsec** | **SD** | **Control group** | **Median stereopsis in logarcsec** | **SD** | **P (difference between groups)** |
| --- | --- | --- | --- | --- | --- | --- | --- |
| Homes et al | 41 | 0.22 |  | 39 | 0 |  | Not reported |
| Dahlmann-Noor et al. | 16 | No significant difference reported |  | 16 | No significant difference reported |  | Not significant difference reported |
| Chen et al. | 20 | 0.95 Mean | 0.76 | 22 | 0.82 Mean | 0.59 | 0.712 |
| Handa et al | 20 | Not reported |  | 20 | Not reported |  |  |
| Roy et al. | 27 | 0.10  Mean value reported | 0.16 | 28 | 0.003  Mean value reported | 0.17 | 0.7 |
| Iwata et al. | 23 | Not reported | - | 23 | Not reported | - | - |
| Kadhum et al. | 16 | 1.40 |  | 17 | 1.40 |  | 0.609 |

**S12 Table. Search Terms. Summary of findings.**

**Digital binocular therapy compared with conventional patching for unilateral amblyopia in treatment-naive children**

**Population:** Children aged 3–13 years with unilateral amblyopia after refractive adaptation
**Intervention:** Digital binocular therapy
**Comparison:** Conventional patching

| **Outcomes** | **Assumed risk (Patching)** | **Corresponding risk (Digital binocular therapy)** | **Relative effect (95% CI)** | **No. of participants (studies)** | **Certainty of the evidence (GRADE)** | **Comments** |
| --- | --- | --- | --- | --- | --- | --- |
| **Change in visual acuity (logMAR)** | Not applicable | Not applicable | MD 0.00 (−0.09 to 0.10) | 225 (5 RCTs) | ⊕⊕⊕◯ Moderate¹ | No clinically meaningful difference. |
| **Stereopsis** | Not estimable | Not estimable | Not pooled | 225 (5 RCTs) | ⊕◯◯◯ Very low²³ | No consistent between-group differences reported. |
| **Adherence** | Not estimable | Not estimable | Not pooled | 225 (5 RCTs) | ⊕◯◯◯ Very low²³ | Variable adherence; age-related differences observed. |
| **Adverse effects** | Rare events | Rare events | Not pooled | 225 (5 RCTs) | ⊕◯◯◯ Very low²³ | Transient headache/diplopia reported in one study. |

**Explanations**

1. Downgraded one level for **inconsistency** (moderate heterogeneity, variable treatment effects and wide prediction interval).
2. Downgraded one level for **inconsistency** (no quantitative synthesis was possible due to heterogeneity in outcome measurement and reporting, and study findings varied in direction and magnitude of effects).
3. Downgraded two levels for **very serious imprecision** (small sample sizes, lack of confidence intervals and uncertain effect estimates).

**S13 Table. PRISMA 2020 checklist**

| **Section and Topic** | **Item #** | **Checklist item** | **Location where item is reported** |
| --- | --- | --- | --- |
| **TITLE** | | |  |
| Title | 1 | Systematic review  Evaluation of digital binocular therapies for amblyopia after refractive adaptation in treatment-naive children aged 3–13 years: a systematic review and meta-analysis of randomised controlled trials. | Page 1  Lines 1-4 |
| **ABSTRACT** | | |  |
| Abstract | 2 | A structured summary including: background; objectives; design; results; conclusion; and implications of key findings with limitations. | Page 2-3  Lines 26-54 |
| **INTRODUCTION** | | |  |
| Rationale | 3 | Amblyopia management traditionally relies on refractive adaptation followed by patching. However, patching faces many challenges like poor compliance, social stigma, and failure to address interocular binocular function. While digital binocular therapies have emerged to promote balanced binocular input and improve engagement, existing systematic reviews have yielded inconsistent results.  A primary limitation of previous reviews is the remarkable population heterogeneity. Most of the reviews included children or adults who had undergone patching followed by binocular treatments which could mask the true efficacy of binocular interventions. | Page 3-4  Lines 68-71, 77-80, 96-97 |
| Objectives | 4 | The objective of this systematic review and meta-analysis is to evaluate the effectiveness and efficacy of digital binocular therapies (including games, virtual reality, or movies) compared to conventional patching therapy for improving best-corrected visual acuity (BCVA) in treatment-naive children aged 3–13 years with unilateral amblyopia, following a completed period of refractive adaptation. By restricting this review to children who have only undergone refractive adaptation and have no prior history of patching, this study addresses a critical evidence gap to determine if digital binocular therapies can serve as an effective first-line alternative to conventional treatment.  The review particularly addresses whether these digital platforms can serve as a viable primary intervention in clinical practice. | Page 5  Lines 99-105 |
| **METHODS** | | |  |
| Eligibility criteria | 5 | Eligibility Criteria  The research question was formulated using the Population, Intervention, Comparator, Outcome (PICO) framework recommended in the Cochrane Handbook for Systematic Reviews of Interventions.  Population:  Children (3–13 years) with unilateral amblyopia following a documented period of refractive adaptation. Only treatment-naive participants were included.  Intervention:  Digital binocular therapy including dichoptic therapy delivered via gamified platforms such as tablets, virtual reality or computers utilising different visual stimuli to each eye to promote binocular interaction and reduce suppression.  Comparator:  Refractive adaptation (spectacle correction alone) and/or conventional occlusion therapy (patching).  Outcomes:  Primary outcome: Change in visual acuity measured using standardised logMAR charts (e.g., ATS HOTV, E-ETDRS).  Secondary outcomes: Stereoacuity, treatment adherence, and adverse events.  Inclusion and Exclusion Criteria  Eligible studies were RCTs published in peer-reviewed journals between January 2000 and December 2025, involving children with newly diagnosed amblyopia, who had completed a documented period of refractive adaptation (at least 16–18 weeks or until stable visual acuity as defined within individual trials) and not received prior amblyopia treatment other than spectacle adaptation. Only full-text articles published in English were included to ensure accurate extraction of complex clinical and statistical data, although this may introduce language bias. Studies were excluded if they were pilot studies without subsequent follow-up trials, review articles, non-randomised studies, trials without a control group, or interventions involving non-digital therapies [other than spectacles and/or patching]. Perceptual learning methods, transcranial magnetic stimulation, acupuncture, purely classical therapies (patching or atropine alone), and combination therapies where crossover prevented estimation of independent treatment effects were also excluded. | Page 5-7  Lines 117-154 |
| Information sources | 6 | Medline (Ovid), Scopus, Web of Science, Embase, PubMed, Cochrane Library, and Google Scholar. Additional sources include Joanna Briggs Institute, NICE, Prospero, SIGN, Trip Database, Centre for Evidence-Based Medicine, and clinical trial registries. Hand-searching and forward/backward reference chaining were performed. Grey literature was explored through open-access university repositories.  A prior search strategy from an earlier version of the review was adapted, refined, and updated to align with the objectives of the current study.  Searches were conducted from database inception to 31 December 2025. | Page 7  Lines 156-167 |
| Search strategy | 7 | S1 \| Amblyopi*; S2 \| Child* OR pediatric* OR paediatric*; S3 \| Dichoptic*; S4 \| “digital* ther*”; S5 \| Digital NEXT ther*; S6 \| “comput* gam*”; S7 \| Comput* gam*; S8 \| S1 AND S2 AND S3; S9 \| S1 AND S2 AND S4; S10 \| S1 AND S2 AND S6; S11 \| S8 OR S9 OR S10; S12 \| Amblyopi* AND video*; S13 \| Amblyopi* AND IPAD; S14 \| Amblyopi* AND television OR TV; S15 \| Amblyopi* AND movi*; S16 \| Amblyopi* AND occlu-pad or occlu-tab; S17 \| Amblyopi* AND naive; S18 \| S12 OR S13 OR S14 OR S15 OR S16 OR S17; S19 \| S11 AND S18 Electronic databases were searched from January 2000 to December 2025 using combinations of keywords and subject headings related to amblyopia, children, and digital binocular therapies, including dichoptic therapy, digital therapeutics, computer games, video-based interventions, and device-based treatments (e.g., Occlu-pad). Searches were limited to randomized controlled trials published in English. | Supplementary Tables S1–S2. |
| Selection process | 8 | The full texts were screened independently by two reviewers. Data were extracted using a standardised form by the same reviewers. Disagreements at any stage were resolved through discussion or arbitration by a third reviewer. No automation tools or machine-learning software were used during the screening or study selection process. | Page 7-8  Lines 171-176 |
| Data collection process | 9 | Data were extracted independently by two reviewers using a standardized data extraction form. Extracted information included study characteristics, participant demographics, intervention and comparator details, outcome measures, follow-up duration, and all outcomes relevant to the review. After independent extraction, the reviewers compared their data for accuracy and completeness, and any discrepancies were resolved through discussion. Where necessary, corresponding authors were contacted to obtain missing information or to clarify unclear or incomplete data. No automation tools were used in the data extraction process. | Page 8  Figure 1  Lines 171-182 |
| Data items | 10a | The primary outcome was best-corrected visual acuity (BCVA), measured in logarithm of the minimum angle of resolution (logMAR) units. Secondary outcomes included stereoacuity, treatment adherence or compliance, and occurrence of adverse events. Data at the end of the intervention period were prioritized for the primary analysis.  For each trial, the number of participants randomised at baseline was recorded. For meta-analysis, the effective sample size was defined as the number with available follow-up outcome data. Where intention-to-treat data were unavailable, per-protocol data from participants completing the intervention and outcome assessment were used.  Attrition rates and reasons were considered during the risk of bias assessment for incomplete outcome data. When continuous outcomes were reported as medians and interquartile ranges rather than means and standard deviations, the median was treated as an approximation of the mean, and the standard deviation was estimated using the formula SD ≈ IQR/1.35. When SD of change in VA was not reported, the Cochrane‑recommended formula, assuming a correlation coefficient of r=0.5 was used. | Page 6, 8,  Lines 128-131, 184-195,  Table 1  Supplementary Tables S3 and S4 |
|  | 10b | Additional data extracted included country, study design, clinical setting, sample size, age, sex, type and severity of amblyopia, baseline visual acuity, prior treatment history, and whether participants were treatment-naive following refractive adaptation only, and intervention characteristics including type of digital binocular therapy, treatment platform, dosage, frequency, duration, and comparator intervention). Information on follow-up duration, treatment adherence, attrition rates, funding sources, and declarations of conflicts of interest was also collected.  Where data were missing, unclear, or incompletely reported, the corresponding authors were contacted for clarification and if no response was received, studies were excluded without relevant data. | Table 1  Page 7  Lines 153-154  Supplementary Table S5 and S6 |
| Study risk of bias assessment | 11 | Risk of bias was assessed independently by two reviewers using the Cochrane Risk of Bias 2 (RoB 2) tool for randomized controlled trials. The domains evaluated included bias arising from the randomization process, deviations from intended interventions; missing outcome data; bias in the measurement of outcomes and bias in reported result. Each domain was judged as having low risk of bias, some concerns, or high risk of bias, in accordance with the RoB 2 guidance.  Any disagreements between reviewers were resolved through discussion and consensus, with consultation from a third reviewer when necessary. Domain-level judgments were used to determine an overall risk of bias rating for each study. No automation tools or software-assisted risk of bias assessment methods were used. | Page 9  Lines 199-209  Supplementary Table S7 - 10 |
| Effect measures | 12 | For the primary outcome of best-corrected visual acuity, measured in logMAR units, treatment effects were summarized using mean differences and if not given it was calculated from the final and initial visual acuity. A negative mean difference indicated greater improvement in visual acuity in the digital binocular therapy group compared with the control group.  For secondary continuous outcomes (primarily stereoacuity), as there were no comparable scales, quantitative synthesis was not appropriate, and findings were summarized narratively. Other secondary outcomes, including adherence and adverse events, were reported inconsistently and with insufficient quantitative data for pooling, these outcomes were narratively summarised. | Page 6, 10  Lines 128-131, 223-244  Final Data List Table S8,  Table S11 |
|  | 13a | Eligibility criteria of the studies were based on the prespecified inclusion criteria relating to population, intervention, comparator, and outcomes and also risk of bias against the pre-specified PICO criteria and the planned primary vs sensitivity analyses. Studies that met the eligibility criteria but were judged to be at high risk of bias were excluded from the primary meta-analysis and GRADE assessment, although they were included in a predefined sensitivity analysis. Secondary outcomes, including stereoacuity, adherence, and adverse events, were synthesized narratively when data were insufficient, inconsistently reported, or too heterogeneous for quantitative pooling. | Page 5-6, 9, 10  Lines 117-144, 201-208, 233-244, 254-261 |
|  | 13b | Prior to synthesis, data were checked for consistency, completeness, and compatibility across studies. As all included studies reported best-corrected visual acuity in logMAR units, no unit conversions were required for the primary outcome.  For each trial, the number of participants randomised at baseline was recorded. For meta-analysis, the effective sample size was defined as the number with available follow-up outcome data. Where intention-to-treat data were unavailable, per-protocol data from participants completing the intervention and outcome assessment were used. Attrition rates and reasons for dropout were documented and considered in the risk of bias assessment.  When outcomes were reported as median and interquartile range (IQR) rather than mean and standard deviation (SD), the median was used to approximate the mean, and the SD was estimated as IQR/1.35 assuming a normal distribution. When SD of change in visual acuity was not reported, the Cochrane-recommended formula, assuming a correlation coefficient of r = 0.5, was used. | Page 8  Lines 185-195 |
|  | 13c | Results of individual studies and the pooled syntheses were displayed in forest plots generated with the meta package in R. Study characteristics and secondary outcomes were summarised in tables. | Figure 2 and 3  Table 1 |
|  | 13d | Mean differences (MD) in logMAR visual acuity (change from baseline) were pooled using both fixed-effect and random-effects models. Statistical heterogeneity was assessed using the I² statistic and τ². Analyses were performed in R (version 4.6.0) within RStudio using the meta package. Random-effects results were considered the primary estimates because of expected clinical and methodological diversity. Secondary outcomes (stereoacuity, adherence, adverse events) could not be pooled due to inconsistent measurement and reporting; these were summarised narratively following the SWiM framework. | Page 10  Lines 223-244 |
|  | 13e | Formal subgroup analyses and meta-regression were not performed because of the small number of included studies (n = 5 in the primary analysis). | Page 10  Lines 229-232 |
|  | 13f | Two high risk studies were added to the included studies for pre specified sensitivity analysis. This analysed if the pooled effect estimates or the overall interpretation of findings changed. | Page 9  Lines 05-208 |
| Reporting bias assessment | 14 | Risk of bias due to missing results arising from reporting biases, including publication bias, was considered for the primary outcome. However, formal assessment using funnel plots was not undertaken because fewer than 10 studies were included in the meta-analysis, making such methods unreliable and potentially misleading. Consequently, publication bias could not be formally assessed and was considered unclear. This limitation was taken into account when interpreting the overall certainty of the evidence. | Page 23  Lines 497-500 |
| Certainty assessment | 15 | The certainty of evidence for each outcome was assessed using the Grading of Recommendations Assessment, Development and Evaluation (GRADE) approach. RCTs started as high-certainty evidence and were evaluated across five domains (risk of bias, inconsistency, indirectness, imprecision, and publication bias).  Downgrading for quantitative outcomes was guided by pooled estimates, confidence intervals, heterogeneity, and risk-of-bias assessments; for narrative outcomes, particular attention was given to inconsistency, measurement variability, sparse data, and imprecision. Publication bias could not be formally assessed (<10 studies). Assessments were performed independently by two reviewers with consensus resolution. Overall ratings appear in the Summary of Findings table; full domain-level judgements are in the supplementary materials. | Page 11, 20-21  Lines 211-221, 433-440, 458-464  Table 2  Supplementary Table S10 |
| **RESULTS** | | |  |
| Study selection | 16a | A total of 1030 records were identified from databases (n=804), trial registers (n=125), and other sources (101). Following the removal of 549 duplicates, 481 records underwent title screening. Of these, 308 were excluded at title level, leaving 173 records for abstract screening. After abstract review, 154 reports were excluded, and 19 full-text reports were assessed for eligibility. Ultimately, 14 reports were excluded for the following reasons: combination therapy (n=1), ongoing trial (n=1), abstract-only availability (n=1), previous therapy (n=5), high risk of bias (n=2), full text unavailable after contacting author (n=1), and single-arm study (n=3). Five studies met all inclusion criteria (5 RCTs; n=225) and were included in the primary meta-analysis and GRADE assessment. Two additional studies (7 RCTs; n = 292), that otherwise met the eligibility criteria but were judged to be at high risk of bias were included only in the predefined sensitivity analysis. | Page 11  Lines 248-258  Figure 1 |
|  | 16b | Fourteen full-text reports were excluded after detailed assessment against the prespecified eligibility criteria. Reasons for exclusion included combination therapy (n = 1), ongoing trial (n = 1), abstract-only publication (n = 1), prior amblyopia treatment other than refractive adaptation (n = 5), high risk of bias (n = 2), full text unavailable despite attempts to contact the corresponding author (n = 1), and single-arm study design (n = 3).  Two studies that otherwise met the eligibility criteria were excluded from the primary meta-analysis and GRADE assessment because they were judged to be at high risk of bias and included in sensitivity analysis. Summaries of all excluded studies are provided in the supplementary materials, along with detailed domain-level risk of bias assessments. | Page 11, 15-16  Lines 254-258, 321-339  Supplementary Table S18 |
| Study characteristics | 17 | Five randomized controlled trials met the inclusion criteria: Holmes et al. [42], Dahlmann-Noor et al. [43], Chen et al. [44], Handa et al [45], and Roy et al. [32] Together, these studies included 225 children aged 3–13 years with unilateral amblyopia following refractive adaptation.  All included studies enrolled treatment-naive children who had completed refractive adaptation and had received no prior amblyopia treatment other than refractive correction. The interventions evaluated were digital binocular therapies, including dichoptic games, balanced binocular viewing, smartphone-based dichoptic training, and the Occlu-tab platform. Comparator interventions included conventional monocular patching or continued refractive correction alone.  Holmes et al. [42] compared an iPad-based anti-suppression game with patching in young children. Dahlmann-Noor et al. [43] evaluated balanced binocular viewing using modified movies delivered through LCD shutter glasses. Chen et al. [44] assessed a home-based dichoptic iPad game compared with patching. Handa et al. [45] investigated the Occlu-tab device in comparison with patching, while Roy et al. [32] evaluated smartphone-based dichoptic therapy against conventional patching.  Across all studies, the primary outcome was best-corrected visual acuity measured in logMAR units. Secondary outcomes included stereoacuity, adherence or compliance, and adverse events, although these were reported inconsistently across trials. Study settings varied, including home-based, clinic-based, and hybrid delivery models. Holmes et al. [42] was conducted in clinical or hospital-based settings, Dahlmann-Noor et al. [43], Chen et al. [44], and Roy et al. [32], were home-based, and Handa et al. [45] used a hybrid model combining clinic-based and home-based training.  The study by Roy et al. [32] was for children aged 5-15 years, the trial was retained in primary analysis, because the subgroup represented only a small proportion of the total analysed population (24/225; 10.7%) and the overall study population remained broadly consistent with the target population according to GRADE and Cochrane guidelines.  Detailed characteristics of the included studies, including their methodological strengths and limitations, are presented in supplementary data. | Page 11-12, 22  Lines 266-295, 472-485  Table1  Supplementary tables S6 – S10 |
| Risk of bias in studies | 18 | Overall, the five included RCTs were judged to have a low risk of bias or some concerns across most domains using the Cochrane RoB 2 tool. Lack of blinding was inherent to the intervention design because of the active nature of digital interventions, resulting in some concerns in the domain of deviations from intended interventions (Domain 2). Potential differences in adherence across groups may also have influenced outcomes. However, the risk of bias in other domains remained low, as outcome assessment was generally standardised and evidence of selective reporting bias was minimal. Although masking of participants and personnel was not possible, the primary outcome (logMAR visual acuity) was measured objectively by masked assessors using standardised charts; therefore, the risk of bias was not considered serious enough to warrant downgrading the certainty of evidence.  Individually, Holmes et al. [42], Dahlmann-Noor et al. [43], Chen et al. [44], and Roy et al. [32] were judged to have some concerns overall, primarily due to lack of blinding of participants and personnel. Handa et al. [45] was judged to have low risk of bias across all domains. Two additional studies (Iwata et al. [41] and Kadhum et al. [42]) were judged to be at high risk of bias and were therefore excluded from the primary analysis but retained for sensitivity analysis. Detailed domain-level risk of bias assessments are presented in Appendix V (Supplementary Tables S11 and S12). | Page 15-16  Lines 305-341  Supplementary Table S7 – S10 |
| Results of individual studies | 19 | For the primary outcome of best-corrected visual acuity, all five included studies reported mean changes in logMAR visual acuity from baseline. Holmes et al., Dahlmann-Noor et al., Chen et al., and Roy et al. demonstrated similar improvements in visual acuity in the digital binocular therapy and control groups. In contrast, Handa et al. reported statistically significant improvement in binocular group at the end of follow-up at 8 weeks.  Study-specific treatment effects, expressed as mean differences (MDs) with 95% confidence intervals (CIs), are presented in the forest plots for the primary meta-analysis (Figure 2) and sensitivity analysis (Figure 3).  For secondary outcomes, stereoacuity findings were inconsistent across studies. Holmes et al. reported negligible median changes (binocular 0.22 vs patching 0 log arcsec), Dahlmann-Noor et al. reported no significant change, Chen et al. found no between-group difference (0.95 vs 0.82 log arcsec; p=0.71), Handa et al. did not report stereoacuity outcomes, and Roy et al. reported no significant difference (p=0.07).  Adherence or compliance outcomes were reported inconsistently. Holmes et al. did not report adherence for the relevant cohort. Dahlmann-Noor et al. reported moderate adherence to binocular therapy (mean 56–58%). Chen et al. reported better adherence with binocular therapy (median deviation −1.7% for binocular therapy versus −13.7% for patching); however, adherence was measured objectively only in the binocular arm, whereas patching adherence was self-reported. Handa et al. reported full compliance in both groups, and Roy et al. reported better adherence in the patching group.  Adverse events were generally mild and uncommon. Holmes et al. reported rare diplopia and worsening of <10% new-onset heterotropia in both groups. Dahlmann-Noor et al. reported headaches and withdrawal of two participants. Chen et al. and Handa et al. reported no serious adverse events, while Roy et al. reported transient adverse events in the dichoptic therapy group. | Page 16-17, 18-20  Lines 351-362, 388-433  Figure 2, 3  Supplementary Table S6 |
| Results of syntheses | 20a | The primary analysis included 5 RCTs involving 225 treatment-naïve children aged 3–13 years with unilateral amblyopia following refractive adaptation and compared digital binocular therapy with conventional patching or refractive correction alone. Interventions included dichoptic games, balanced binocular viewing, smartphone-based dichoptic training, and the Occlu-tab platform. Overall, the contributing studies were judged to be at low risk of bias or to raise only some concerns, primarily due to the unavoidable lack of participant masking.  The sensitivity analysis included two additional randomized trials (7RCTs; n=292) that otherwise met the eligibility criteria but were judged to be at high risk of bias. These studies were excluded from the primary synthesis because of important methodological limitations, including inadequate allocation concealment, lack of masking, high attrition, use of non-standard visual acuity assessment methods, and reliance on self-reported adherence. Inclusion of these studies increased statistical heterogeneity substantially.  The secondary outcomes, including stereoacuity, adherence, and adverse events were assessed from the five included trials. Reporting was inconsistent, and outcome measures varied across studies. The certainty of evidence was reduced by methodological limitations related to lack of masking, incomplete outcome reporting, and imprecision arising from small sample sizes and sparse data. Consequently, these outcomes were synthesized narratively rather than quantitatively. | Page 17-20  Lines 368-443  Table 2 |
|  | 20b | Meta-analysis was conducted only for the primary outcome of best-corrected visual acuity. Across five randomized controlled trials involving 225 children, there was no statistically significant difference between digital binocular therapy and conventional patching at the end of treatment.  The common/fixed-effect model showed a mean difference (MD) of −0.01 logMAR (95% CI −0.04 to 0.02), while the random-effects model showed an MD of 0.00 logMAR (95% CI −0.09 to 0.10). Both models therefore indicate no clear advantage of digital binocular therapy over patching. Moderate heterogeneity was observed (I²=50.2%; τ²=0.0023; p=0.09), suggesting moderate variability between studies, although the estimated between-study variance was low. The 95% prediction interval (−0.15 to 0.16 logMAR) crossed zero and extended beyond the clinically meaningful threshold of ±0.1 logMAR, indicating that a future study could potentially show a clinically important advantage for either intervention.  A sensitivity analysis incorporating two additional studies at high risk of bias (seven trials; 292 participants) yielded similar findings. The random-effects model showed no statistically significant difference between digital binocular therapies and patching for end-of-treatment visual acuity (MD 0.04 logMAR, 95% CI −0.03 to 0.10). Although the fixed-effect model suggested a small advantage for patching (MD 0.03 logMAR, 95% CI 0.00 to 0.06), this finding was not robust under random-effects modelling and should be interpreted cautiously given the substantial heterogeneity (I²=75.2%; τ²=0.0046; heterogeneity p=0.0005). The 95% prediction interval (−0.15 to 0.22 logMAR) was wide and crossed zero, indicating that the true treatment effect could favour either intervention. Overall, there was no consistent evidence that digital binocular therapies are superior to patching.  No statistical meta-analyses were performed for secondary outcomes, including stereoacuity, adherence or compliance, and adverse events, because of substantial heterogeneity in measurement methods, reporting formats, and completeness of data. These outcomes were synthesised narratively. Across the included studies, stereoacuity showed no consistent between-group differences. Adherence findings were mixed, with some studies suggesting better engagement with digital therapy, although measurement methods varied considerably. Adverse events were rare, mild, and transient, with no serious safety concerns reported.  Because the pooled effect estimate was very close to zero and the 95% confidence interval crossed the line of no effect, there is no evidence that digital binocular therapy is superior to conventional patching for improving visual acuity following refractive adaptation in treatment-naïve children with amblyopia. | Page 16-20  Lines 351-434  Figure 2, 3 |
|  | 20c | Moderate heterogeneity was observed in the primary analysis (I² = 50.2%) and increased to substantial heterogeneity in the sensitivity analysis after inclusion of two studies at high risk of bias (I² = 75.2%). Formal subgroup analyses were not performed because of the small number of included studies. Clinical differences across studies, including participant age, amblyopia type, treatment dosage, and adherence, were considered as potential contributors to heterogeneity. The heterogeneity contributed to downgrading the certainty of evidence for visual acuity to moderate, though the overall conclusion remained unchanged. | Page 10, 17, 22, 24  Lines 229-231,  356-361, 376-379, 492-494, 528-531  Table 2 |
|  | 20d | Moderate heterogeneity was observed in the primary analysis (I² = 50.2%) increased to substantial heterogeneity in the sensitivity analysis (I² = 75.2%) after inclusion of high-risk-of-bias studies. However, pooled estimates remained close to no effect under random-effects modelling (primary: MD 0.00, 95% CI −0.09 to 0.10; sensitivity: MD 0.04, 95% CI −0.03 to 0.10). Wide confidence intervals and prediction intervals (primary: −0.15 to 0.16; sensitivity: −0.15 to 0.22) indicated considerable uncertainty, with no reliable evidence favouring digital binocular therapies or patching for improving visual acuity in children with unilateral amblyopia. | Page 16 -18  Lines 351-362, 369-382  Figure 2, 3 |
| Reporting biases | 21 | Publication bias could not be formally assessed because fewer than 10 studies were included in the meta-analysis rendering formal statistical tests underpowered (n=5 in the primary analysis; n=7 in the sensitivity analysis). Therefore, the risk of reporting bias remains unclear. For the secondary outcomes, missing or incompletely reported data were described in the narrative synthesis and considered when interpreting the findings. | Page 18, 23  Lines 389-398, 498-501  Table 2  Supplementary Tables S7 – SS10 |
| Certainty of evidence | 22 | The GRADE framework was used to assess certainty for all outcomes. Visual acuity was rated as moderate certainty (downgraded one level for inconsistency: $I^{2}=50.2\%$(I² = 50.2%). Stereoacuity, adherence, and adverse events were rated as very low certainty (downgraded for inconsistency and very serious imprecision). | Page 22-23  Line 492-500  Table 2 |
| **DISCUSSION** | | |  |
| Discussion | 23a | In treatment-naive children with unilateral amblyopia who had received only spectacle correction, digital binocular therapies demonstrated no clinically meaningful difference in visual acuity compared to patching. There is no clear superiority of digital binocular therapy over patching. Although these interventions are designed to target binocular mechanisms such as interocular suppression, this theoretical advantage has not yet translated into superior clinical outcomes, particularly for visual acuity or stereoacuity. Digital binocular therapies may serve as promising adjunctive or alternative options for children with poor adherence to occlusion therapy. Further high-quality trials are needed to determine whether advances in digital treatment platforms can yield clinically meaningful benefits. The certainty of evidence was low mainly because of inconsistency and imprecision.  To our knowledge, no previous systematic review has specifically evaluated digital binocular therapies in treatment-naive children following refractive adaptation; therefore, direct comparison with previous reviews in this population is not possible. We could only broadly compare our findings with previous systematic reviews and meta-analyses that included mixed populations of treatment-naive and previously treated children. | Page 24-26  Lines 522-548, 570-580 |
|  | 23b | The evidence included in this review has several limitations. Only five eligible randomised controlled trials were identified, with a modest overall sample size (n = 225), limiting the precision of the effect estimates. Two studies judged at high risk of bias were excluded from the primary analysis and included only in a sensitivity analysis. Moderate heterogeneity was observed in the primary analysis (I² = 50.2%), increasing substantially in the sensitivity analysis (I² = 75.2%), likely reflecting differences in participant age, amblyopia subtype, digital treatment platforms, and prescribed treatment dosage. These limitations reduce confidence in the overall findings, particularly for secondary outcomes. | Page 27  Lines 598-613 |
|  | 23c | Restricting inclusion to studies published in English may have introduced language bias. The small number of included studies limited the ability to formally assess publication bias using funnel plots. The relatively small number of available and included high-quality studies highlights the lack of published literature in this area. This has a bearing on the power of this study to quantify the true effect of digital binocular therapies. However, it also highlights an obvious gap in the existing evidence base. Meta-analysis was not possible for secondary outcomes because of inconsistent outcome measurement and reporting across studies requiring narrative synthesis. Despite these limitations, the review followed a pre-specified protocol, employed a comprehensive search strategy, and used independent duplicate screening, data extraction, and risk-of-bias assessment to minimise potential bias in the review process. | Page 27  Lines 519-614 |
|  | 23d | Current evidence does not support replacing conventional patching with digital binocular therapies as first-line treatment for unilateral amblyopia in treatment-naive children following refractive adaptation. The digital binocular therapies may be considered as adjunctive or alternative options in situations where adherence to patching is poor or treatment engagement is challenging.  Implementation of digital binocular therapies requires stronger evidence from adequately powered, high-quality RCTs, in treatment-naive population.  The future trials should incorporate standardised outcome measures, objective adherence monitoring, systematic reporting of adverse events, and longer follow-up to determine whether digital binocular therapies can provide meaningful advantages in visual and binocular outcomes over conventional treatment. | Page 27-28  Lines 616-628 |
| **OTHER INFORMATION** | | |  |
| Registration and protocol | 24a | This systematic review was prospectively registered with PROSPERO CRD420261278100. | Page 5, 29  Lines 109-111, 665-666 |
|  | 24b | The review protocol is available in PROSPERO CRD420261278100. | Page 5, 29  Lines 109-111, 665-666 |
|  | 24c | No substantive amendments were made to the review methods or outcomes after registration in PROSPERO. | Page 5, 29  Lines 109-111 |
| Support | 25 | None, no funders or sponsors had any role in the design, conduct, analysis, interpretation, or reporting of this review. | Page 29  Line 653 |
| Competing interests | 26 | The authors declare that they have no competing interests. | Page 29  Line 654 |
| Availability of data, code and other materials | 27 | The extracted study data, data used for all analyses, and other materials relevant to this review are available in the supplementary online files accompanying this article. Additional information may be obtained from the corresponding author upon reasonable request. Analytic code was not generated for this review. | Page 29  Lines 660-664 |
